# METTL16 promotes liver cancer stem cell self-renewal via controlling ribosome biogenesis and mRNA translation

**DOI:** 10.1186/s13045-024-01526-9

**Published:** 2024-02-01

**Authors:** Meilin Xue, Lei Dong, Honghai Zhang, Yangchan Li, Kangqiang Qiu, Zhicong Zhao, Min Gao, Li Han, Anthony K. N. Chan, Wei Li, Keith Leung, Kitty Wang, Sheela Pangeni Pokharel, Ying Qing, Wei Liu, Xueer Wang, Lili Ren, Hongjie Bi, Lu Yang, Chao Shen, Zhenhua Chen, Laleh Melstrom, Hongzhi Li, Nikolai Timchenko, Xiaolan Deng, Wendong Huang, Steven T. Rosen, Jingyan Tian, Lin Xu, Jiajie Diao, Chun-Wei Chen, Jianjun Chen, Baiyong Shen, Hao Chen, Rui Su

**Affiliations:** 1 Department of Systems Biology, Beckman Research Institute of City of Hope, Monrovia, CA 91016 USA; 2 City of Hope Comprehensive Cancer Center, City of Hope, Duarte, CA 91010 USA; 3 Gehr Family Center for Leukemia Research, City of Hope, Duarte, CA 91010 USA; 4 Department of General Surgery, Pancreatic Disease Center, Ruijin Hospital, Shanghai Jiao Tong University School of Medicine, Shanghai, 200025 China; 5 Department of Diabetes Complications and Metabolism, Diabetes and Metabolism Research Institute, Beckman Research Institute of City of Hope, Duarte, CA 91010 USA; 6 Graduate School of Biological Science, City of Hope, Duarte, CA 91010 USA; 7 Department of Cancer Biology, University of Cincinnati College of Medicine, Cincinnati, OH 45267 USA; 8 Department of Radiation Oncology, The First Affiliated Hospital of Sun Yat-Sen University, Guangzhou, 510080 Guangdong China; 9 Department of Liver Surgery, Renji Hospital, School of Medicine, Shanghai Jiao Tong University, Shanghai, 200127 China; 10 School of Pharmacy, China Medical University, Shenyang, 110001 Liaoning China; 11 Division of Surgical Oncology, Department of Surgery, Beckman Research Institute of City of Hope Comprehensive Cancer Center, Duarte, CA 91010 USA; 12 Department of Molecular Medicine, City of Hope National Medical Center, Duarte, CA 91016 USA; 13 Division of General and Thoracic Surgery, Cincinnati Children’s Hospital Medical Center, Cincinnati, OH 45229 USA; 14 Quantitative Biomedical Research Center, Peter O’Donnell Jr. School of Public Health, UT Southwestern Medical Center, Dallas, TX 7539 USA; 15 State Key Laboratory of Medical Genomics, Clinical Trial Center, Shanghai Institute of Endocrine and Metabolic Diseases, Department of Endocrinology and Metabolism, Ruijin Hospital, Shanghai Jiao Tong University School of Medicine, Shanghai, 200025 China

**Keywords:** METTL16, N^6^-methyladenosine, Cancer stem cells, Self-renewal, Hepatocellular carcinoma, Ribosome biogenesis, mRNA translation, eIF3a

## Abstract

**Background:**

While liver cancer stem cells (CSCs) play a crucial role in hepatocellular carcinoma (HCC) initiation, progression, recurrence, and treatment resistance, the mechanism underlying liver CSC self-renewal remains elusive. We aim to characterize the role of Methyltransferase 16 (METTL16), a recently identified RNA *N*^6^-methyladenosine (m^6^A) methyltransferase, in HCC development/maintenance, CSC stemness, as well as normal hepatogenesis.

**Methods:**

Liver-specific *Mettl16* conditional KO (cKO) mice were generated to assess its role in HCC pathogenesis and normal hepatogenesis. Hydrodynamic tail-vein injection (HDTVi)-induced de novo hepatocarcinogenesis and xenograft models were utilized to determine the role of METTL16 in HCC initiation and progression. A limiting dilution assay was utilized to evaluate CSC frequency. Functionally essential targets were revealed via integrative analysis of multi-omics data, including RNA-seq, RNA immunoprecipitation (RIP)-seq, and ribosome profiling.

**Results:**

METTL16 is highly expressed in liver CSCs and its depletion dramatically decreased CSC frequency in vitro and in vivo. *Mettl16* KO significantly attenuated HCC initiation and progression, yet only slightly influenced normal hepatogenesis. Mechanistic studies, including high-throughput sequencing, unveiled METTL16 as a key regulator of ribosomal RNA (rRNA) maturation and mRNA translation and identified eukaryotic translation initiation factor 3 subunit a (*eIF3a*) transcript as a bona-fide target of METTL16 in HCC. In addition, the functionally essential regions of METTL16 were revealed by CRISPR gene tiling scan, which will pave the way for the development of potential inhibitor(s).

**Conclusions:**

Our findings highlight the crucial oncogenic role of METTL16 in promoting HCC pathogenesis and enhancing liver CSC self-renewal through augmenting mRNA translation efficiency.

**Supplementary Information:**

The online version contains supplementary material available at 10.1186/s13045-024-01526-9.

## Introduction

Despite tremendous improvements in clinical therapy over the past four decades, liver cancer continues to be a significant global health burden with its prevalence increasing steadily across the world [1]. Hepatocellular carcinoma (HCC) is the most common primary liver cancer and the fourth leading cause of cancer-related deaths worldwide [2]. Due to its high incidence, frequent recurrence, high mortality rates, and limited efficacy of current treatment regimens, HCC represents a major public health concern worldwide. Emerging evidence highlights the critical role of cancer stem cells (CSCs) in the development, progression, and recurrence of HCC [3–6]. Given their unique stem cell-like properties, including self-renewal and differentiation, CSCs can regenerate all properties of a tumor, leading to high heterogeneity and treatment resistance in HCC patients. Targeting liver CSC might be a new approach against HCC.

Dysregulation of messenger RNA (mRNA) translation has been recognized as a hallmark of HCCs and CSCs because elevated protein synthesis is required to sustain neoplastic growth [7, 8]. Translational control during gene expression will endow cells with the ability to promptly adapt to the capricious microenvironment to induce tumorigenesis. Translational regulation can also increase cancer cell plasticity, giving rise to tumor progression and metastasis. mRNA translation is a highly orchestrated process, involving the complicated interplays between ribosomes, translation factors, tRNAs, and amino acids [9]. Ribosome biogenesis, including RNA polymerase I (Pol I)-mediated rRNA transcription, rRNA maturation, and ribosome assembly, mainly takes place in the nucleolus, the largest non-membrane structure in the nucleus [10]. Increased nucleolar size and number—indicative of hyperactive ribosome biogenesis—are associated with advanced tumor stage and inferior cancer survival [11]. The elevated ribosome quantities are required and sufficient to facilitate oncogenic transformation. The strongest evidence is stemmed from the studies showing that induction of Pol I transcription increases ribosome number and nucleolar size, leading to the malignant phenotype [12]. Protein synthesis is carried out by 80S ribosomes in the cytosol of eukaryotes and consists of four major steps, initiation, elongation, termination, and ribosome recycling. Of the four different steps, translation initiation is generally the rate-limiting event [13]. Eukaryotic translation initiation begins with the cooperative assembly of the 43S preinitiation complex (PIC), composed of the ternary complex, eukaryotic translation initiation factor 3 (eIF3), eIF1, and eIF1a on the 40S subunit [14]. The large multiprotein eIF3 complex serves as a scaffold for initiation complex assembly and triggers many steps of the translation initiation pathway. eIF3 binds to a specific group of mRNAs, such as *JUN*, and drives specialized translation to promote carcinogenesis [15]. In addition, forced expression of eIF3 may induce CSC-like properties via remodeling mRNA translation. Thus, a deeper understanding of the molecular mechanisms underlying translational regulation might help discover promising druggable targets to specifically eradicate cancer cells, especially CSCs.

Evidence is emerging that RNA-binding proteins (RBPs) and RNA modifications play crucial roles in translational regulation. The *N*^6^-methyladenosine (m^6^A) modifications on rRNA have been characterized for three decades. METTL5 is responsible for 18S rRNA m^6^A1832 modification [16], while ZCCHC4 is required for 28S rRNA m^6^A 4220 [17]. Both METTL5 and ZCCHC4 play crucial tumor-promoting roles in HCC via facilitating protein synthesis [18, 19]. In addition, the mRNA m^6^A modifications, mainly deposited by the METTL3-METTL14 complex, can enhance mRNA translation efficiency via specific m^6^A-binding proteins [20–22]. The METTL3-eIF3h interaction was found to promote translation and drive malignant transformation by facilitating mRNA circularization [23]. METTL16 is a recently validated m^6^A RNA methyltransferase independent from the METTL3-METTL14 complex, and it was known to catalyze m^6^A formation on only a few substrate RNAs, such as *MAT2A* mRNA and U6 snRNA [24]. Our previous study showed that, amongst the human METTL family with over 30 members, METTL16 is the most essential one for the survival of cancer cells; in addition, METTL16 directly interacts with rRNAs and translation machinery to enhance translation efficiency [25]. However, the definitive role(s) and functionally essential target(s) of METTL16 remain to be determined in human HCCs. It is also necessary to dissect its function in normal bioprocess and development to comprehensively evaluate whether METTL16 can serve as a good target for cancer therapy.

In the present study, we demonstrate that METTL16 plays a crucial role in promoting HCC initiation, progression, and liver CSC self-renewal. Importantly, neither heterogenous nor homozygous *Mettl16* KO exhibits significant effects on normal hepatogenesis. Mechanistically, we have characterized that a set of genes related to mRNA translation and cancer metabolism are directly regulated by the METTL16 in HCC. Furthermore, we reveal a preferential localization of METTL16 in the nucleolus of HCC cells and its essential role in rRNA maturation, ribosome biogenesis, and mRNA translation. These findings provide novel insights into the mechanism of METTL16-mediated translational control in HCC tumorigenesis and the maintenance of liver CSCs.

## Materials and methods

### *Mettl16 *conditional KO mice and hydrodynamic tail-vein injection (HDTVi) model

C57BL/6 J (CD45.2) background *Mettl16*^*fl/fl*^ mice were obtained from Cyagen. Alb-Cre mice were purchased from the Jackson Laboratory. *Mettl16*^*fl/fl*^ mice were generated by inserting floxed LoxP sites flanking exon 3. *Mettl16*^*fl/fl*^ mice were mated to Alb-Cre transgenic mice to generate liver conditional *Mettl16* knockout mice. For HDTVi, groups of 6–8-week-old *Mettl16*^*fl/*+^ Alb-Cre, *Mettl16*^*fl/fl*^ Alb-Cre and wild-type mice received a mix of 5 μg of pCDH-puro-cMyc (46970, Addgene), 5 μg of pX330-p53 (59910, Addgene) and 2.5 μg of CMV-SB13 Transposase as previously described [26]. The genome editing constructs in sterile saline constituted a total volume of 10% of the mouse body weight were injected into the lateral tail vein of mice in 6–8 s. Mice were euthanized 29 days after injection.

### Cell culture

Human HepG2, HEK293T, CL-48, THLE-2, Hep3B, SNU449, PLC/PRF/5 cell lines were obtained from American Type Culture Collection (ATCC), Huh7 and MHCC97H cell lines were given from Professor Wendong Huang (City of Hope, Duarte, CA). HepG2, HEK293T, Huh7 and MHCC97H were all cultured in DMEM medium (Thermo Fisher Scientific), SNU-449 were all cultured in RPMI-1640 medium (Thermo Fisher Scientific), Hep3B, CL-48 and PLC/PRF/5 were all cultured in EMEM medium (ATCC), THLE-2 were all cultured in BEGM Bullet Kit (Lonza), supplemented with 10% fetal bovine serum (Gemini Bio-Products) and 1% penicillin/streptomycin (Thermo Fisher Scientific) at 37 °C in a 5% CO_2_ humidified incubator. All cell lines were identified by STR cell authentication and routinely tested for mycoplasma contamination using PCR Mycoplasma Detection Kit (G238, Applied Biological Materials).

### Plasmid construction

The plasmids used in this study were constructed by In-Fusion cloning (638916, Takara) according to the manufacturer’s instructions. The wild type and some mutant forms of METTL16, and wild type eIF3a/b-3 × HA were generated and used in our previous study [25]. The other mutant forms of METTL16 were amplified using previous wild-type plasmids with indicated mutations by CloneAmp HiFi PCR Premix (639298, Takara) and constructed by In-Fusion cloning. The CDS of eIF3a and eIF3b with a 3 × Flag tag at the N terminus were amplified using previous plasmids and then subsequently cloned into lentivector-based pSIN4 vectors (modified from 61063, Addgene). The wild-type DDX47, DDX49 and BOP1 with a HA tag at the N terminus were amplified using human cDNA made from HEK293T cell and then subsequently cloned into lentivector-based pMIRNA1 (SBI) vectors. All primers were listed in Additional file 1: Table S1. The plasmids were extracted using IBI Hi-Speed Mini Plasmid Kits (IB47102, IBI Scientific) and validated by sanger sequencing (Eton Bioscience).

### CRISPR/Cas9 based genome-editing

LentiCas9-Blast (52962, Addgene) and lenti-sgRNA hygro (104991, Addgene) vectors were used for CRISPR/Cas9 based genome-editing. First, to generate Cas9 single clones, HepG2, Huh7, and Hep3B were transduced with the lentiCas9-Blast lentivirus to stably overexpress Cas9 proteins, and the positive transduction cells were selected with 20 μg/mL blasticidin (ant-bl-1, Invivogen). Subsequently, those cells were seeded into 96-well plates at a density of 0.5 cell/well and the single clones were selected for further expansion. To further evaluate the editing efficiency of Cas9 protein in each clone, we transduced lentivirus co-expressing red fluorescent protein (RFP) and a sgRNA targeting RFP, as we did previously [25, 27], to assess Cas9 editing efficacy by flow cytometry. The HepG2, Huh7, and Hep3B Cas9 single clones with the highest editing efficiency were selected for specific sgRNA transduction. The lenti-sgRNA hygro vector was digested with BsmB1 (R0739, NEB), purified with Gel Extraction Kit (28706X4, Qiagen), and used in the ligation reaction. All sgRNA sequences used in this manuscript were reported in previous study [25].

### Lentivirus production and transfection

2 μg lentiviral vector containing sequences of interest, together with 0.75 μg pMD2.G (12259, Addgene), and 2.25 μg psPAX2 (12260, Addgene) were co-transfected into a 6-cm dish HEK-293 T cells using X-tremeGENE™ HP DNA Transfection Reagent (6366546001, Sigma-Aldrich). After 24 h, the medium may be replaced with complete DMEM medium. Virus-containing supernatant was collected 48 h and 72 h post transfection and centrifuged for 40 min at 3000 g at 4 °C. After that, viral supernatant was added to target cells in the presence of 8 μg/mL polybrene (H9268, Sigma-Aldrich). 48 h after infection, hygromycin B (ant-hg-1, Invivogen) selection at 1 mg/mL was added to obtain stable cell lines.

### Isolation of liver immune cells

Murine liver immune cells were isolated as the previous description with some modifications [28]. Briefly, single-cell suspensions of tissues were collected by spin down at 400 g for 15 min at 4 °C. After resuspended with 8 ml 5% FBS/PBS, the samples were suspended to 4.5 ml isotonic percoll (P1644, Sigma-Aldrich) in 15 ml tube, followed by spin down at 850 g for 25 min at room temperature. After lysed with ACK LYSING Buffer (VWR) to remove red blood cells, cell pellets were collected for further staining.

### Surface and intracellular staining

For surface staining, human liver cancer cells were washed with ice-cold PBS and stained with anti-CD133 PE (372803, BioLegend), and anti-EpCAM APC (324207, BioLegend). Murine liver immune cells were stained with: (1) anti-CD3 APC (100236, BioLegend), anti-CD4 PE (100512, BioLegend), and anti-CD8a FITC (100706, BioLegend); (2) anti-CD19 PE (12-0193-81, Thermo Fisher Scientific) and anti-B220 APC (17-0452-81, Thermo Fisher Scientific); (3) anti-CD3 APC and anti-NK1.1 PE (12-5941-82, Thermo Fisher Scientific); (4) anti-CD11b Percp-Cy5.5 (Mac1, 45-0112-82, eBioscience) and anti-CD11c PE Cy5 (15-0114-82, eBioscience).

Xenograft liver tumor cells were isolated by Gibco™ Collagenase Type IV (17104019, Thermo Fisher Scientific). For intracellular staining, human liver cancer cells and xenograft liver tumor cells were washed with ice-cold PBS and stained with anti-CD133 PE for 30 min at 4 °C. After washed by PBS, cells were fixed in 4%-paraformaldehyde (158127, Sigma-Aldrich) and incubated at 4 °C for 20 min with rotation, followed by resuspended in 5 × Permeabilization buffer (00–8333-56, eBioscience) and incubated at 4 °C for 30 min. The cells were then re-suspended in 1 × Permeabilization buffer and stained with rabbit anti-human METTL16 (1:100, HPA020352, Millipore Sigma) overnight at 4 °C with rotation. Finally, the cells were stained with goat anti-rabbit IgG (H + L) (Alexa Fluor 488 Conjugate, 4412S, Cell Signaling Technology) for 30 min at room temperature, washed twice with 1 × Permeabilization buffer, and re-suspended in FACS buffer for further analysis.

### Cell proliferation assay

Cell proliferation was assessed using CellTiter 96® Non-Radioactive Cell Proliferation Assay (MTT, G400, Promega) and Cell Counting Kit 8 (CCK8, CK04, Dojindo). The cells were seeded into 96-well plates at the concentration of 4 × 10^3^ cells per well in triplicate in a final volume of 100 μl. Following the manufacturer’s recommendation, cell proliferation was measured at OD 570 nm (MTT) or OD 450 nm (CCK8) using the BioTek Gen5 system (BioTeck, USA) according to the manufacturer's instructions.

### Xenograft model

All animal experiments were compliant with federal and state government guidelines and the Institutional Animal Care and Use Committee (IACUC) protocol approved by City of Hope. All mice were housed on a 12–12 h light–dark cycle with ad libitum food and water. The NOD.Cg-Prkdcscid Il2rgtm1Wjl/SzJ (NSG) mice were purchased from Jackson Laboratory (Jax_005557) and bred at the specific-pathogen-free core facilities of City of Hope. Mice of similar age (6–8 weeks) and matched gender were used and randomly assigned to each group. The Cas9-HepG2 cells was established and then infected with pLenti-sgMETTL16-hygromycin, pLenti-sgeIF3a-hygromycin, pLenti-sgeIF3b-hygromycin or scramble control and selected with 1 mg/ml hygromycin. 1 × 10^6^ control, *METTL16* KO, *eIF3a* KO or *eIF3b* KO HepG2 cells were injected subcutaneously into NSG mice. The sizes of tumors were measured using a caliper and the tumor volume was calculated as (width × width × length/2). Mice were euthanized when the tumor volume exceeded 1500 mm^3^.

### Histopathology analysis and immunohistochemistry (IHC)

The mice were euthanized by CO_2_ inhalation and portions of the indicated organs or tumors were employed to paraffin embedding and H&E staining. IHC staining was performed according to standard protocols. Briefly, the samples were deparaffinized, rehydrated through an ethanol series followed by antigen retrieval with sodium citrate or tris–EDTA buffer according to antibody manufacturer’s instruction. Sections were blocked with 10% FBS in PBS for 60 min at room temperature and were incubated with 3% H_2_O_2_ in methanol for 10 min at room temperature to block endogenous peroxidase and then incubated with anti-METTL16 (1:250, HPA020352, Millipore Sigma), anti-eIF3a antibody (1:200; ab86146, Abcam) or anti-eIF3b antibody (1:200; sc-137214, Santa Cruz Biotechnology). IHC staining was performed with horseradish peroxidase (HRP) conjugates using DAB (550,880, Biosciences) detection. All the slides were captured by a Widefield Zeiss Observer 7 microscope.

### Spheroid formation assay

Spheroid formation assays were performed following previously reported protocol with some modifications [29]. Briefly, liver cancer cells were collected and re-suspended in 300 μl of DMEM/F-12, GlutaMAX™ supplement (10,565,018, Invitrogen) supplemented with 50 μl 3% Methylcellulose (HSC001, R&D Systems), 4 μg/ml insulin (12585014, Invitrogen), B27 (1:50; 17504044, Invitrogen), 20 ng/ml human recombinant EGF (E9644, Sigma-Aldrich), 10 ng/ml human recombinant basic FGF (3718-FB, R&D Systems) in 24-well Ultra-Low Attachment Microplates plates (3473, Corning™) for 5–10 days.

For the passage of spheroids, the primary spheroids were collected and dissociated into single cells using Trypsin–EDTA (0.25%) (25200114, Gibco™). Following dissociation, 10%FBS/DMEM was used to neutralize the reaction, and cells were resuspended in DMEM/F-12 medium with the above supplements for another 5–10 days.

### Limiting dilution assay

For in vivo limiting dilution assay, 1 × 10^3^, 1 × 10^4^, 1 × 10^5^ control or *METTL16* KO HepG2 cells were injected subcutaneously into NSG mice. For in vitro limiting dilution assay, HepG2, Huh7, and Hep3B cells with or without *METTL16*, *eIF3a*, or *eIF3b* depletion were seeded at a density of 5000, 2500, 1250, 625, 312, 156, 78, 39 per 48-well plate. The colonies were cultured for 2 weeks. The frequency of LCSC was determined by ELDA (http://bioinf.wehi.edu.au/software/elda/).

### sgRNA library design and CRISPR domain screen

Guide RNA sequences for targeting the coding regions of human METTL16 (Additional file 1: Table S1) were designed using the Genetic Perturbation Platform (Broad Institute) [30]. Briefly, sgRNA oligonucleotides were synthesized via microarray (CustomArray) and cloned into the ipUSEPR lentiviral sgRNA vector [hU6-driven sgRNA co-expressed with EF-1α-driven red fluorescent protein (RFP) and puromycin-resistance gene] using the BsmBI (NEB) restriction sites. For CRISPR screen, Cas9-expressing single clones were infected with lentiviruses containing the sgRNA library at a multiplicity of infection (MOI) < 0.5. After two days, cells were selected with 2 µg/ml puromycin. Genomic DNA was extracted from each screen on day 0 and day 30. The integrated sgRNA-containing regions were amplified by PCR using primers DCF01 5′-CTTGTGGAAAGGACGAAACACCG-3′ and DCR03 5′-CCTAGGAACAGCGGTTTAAAAAAGC-3′. Amplicon sequencing was performed on an Illumina NextSeq 500 sequencer. To quantify sgRNA reads, 20-nucleotide sequences that matched the sgRNA backbone structure (5′ prime CACCG and 3′ prime GTTT) were extracted from FASTQ files and aligned to the sgRNA sequences of the CRISPR screening library using Bowtie2. The frequency for individual sgRNAs was calculated as the read counts of each sgRNA divided by the total read counts matched to the library. For our CRISPR screening, the CRISPR score was defined as a log10-fold-change in the frequency of individual sgRNAs between the end (day 30) and starting time points (day 0) of the screened samples, calculated using the edgeR R package [31] based on the negative binomial distribution of sgRNA read count data. To obtain a CRISPR scan score over regions with no sgRNA coverage, we interpolated the signal via Gaussian kernel smoothing in R [32]. To map CRISPR scan scores to peptide positions, the average CRISPR scan score over the trinucleotide codons was calculated for each peptide position. The smoothed CRISPR scan score was further normalized by the median CRISPR score of the negative control sgRNA (defined as 0.00) and the median CRISPR score of the positive control sgRNA (defined as − 1.00) within the screen data.

### Co-immunoprecipitation (Co-IP)

HepG2 and Huh7 cells in 10-cm cell-culture dishes at 90% confluency were collected and lysed in 1 ml RIPA buffer (87787, Thermo Fisher Scientific) containing 1 × protease inhibitor cocktail (78438, Thermo Fisher Scientific) and 1 × phosphatase inhibitor cocktail (78426, Thermo Fisher Scientific) for 20 min on ice. The protein-containing supernatants were cleaned by centrifugation at 13,000 g for 20 min at 4 °C. 10% volume of protein lysate was kept as input control and the left lysate was mixed with 25 μl Protein A/G magnetic beads (88803, Thermo Fisher Scientific) and rotated at 4 °C for 1 h to reduce any non-specific binding. Then, the pre-cleared lysate (500–1,000 μg) was incubated with anti-FLAG antibody (F3165, Sigma-Aldrich), anti-HA antibody (51064-2-AP, Proteintech), anti-eIF3a antibody (ab86146, Abcam), anti-eIF3b antibody (sc-137214, Santa Cruz Biotechnology), normal mouse IgG (12–371, Millipore) or normal rabbit IgG (12–370, Millipore) under rotation for 1 h at 4 °C, followed by an overnight incubation with 25 μl of pre-washed Protein A/G magnetic beads under rotation at 4 °C. The proteins were collected by magnetic stand, followed by three times washing with IP washing buffer (10 mM Tris–HCl pH 7.5, 1 mM EDTA, 150 mM NaCl, 1% Triton-X, 0.2 mM sodium orthovanadate) and then detected by Western blotting.

### Protein extraction and western blotting

Cells were harvested and lysed with RIPA buffer (R0278, Sigma-Aldrich) supplemented with 1% protease inhibitor cocktail (78438, Thermo Fisher Scientific) and phosphatase inhibitor cocktail (78426, Thermo Fisher Scientific). Western blotting analysis was performed as previously described [33]. Primary antibodies used in this study include anti-METTL16 (1:1000, HPA020352, Millipore Sigma), anti-METTL16 (1:3000, A304-192A, Bethyl), anti-FLAG (1:2000; F3165, Sigma-Aldrich), anti-HA (1:1000; 51,064-2-AP, Proteintech), anti-eIF3a antibody (1:500; ab86146, Abcam), anti-eIF3b antibody (1:1000; sc-137214, Santa Cruz Biotechnology), anti-eIF3c antibody (1:1000; sc-74507, Santa Cruz Biotechnology), anti-eIF1 antibody (1:1000; 15,276-1-AP, Proteintech), anti-eIF2a antibody (1:1000; 11,170-1-AP, Proteintech), anti-eIF4a3 antibody (1:1000; 17,504-1-AP, Proteintech), anti-Puromycin (1:5000; MABE343, Millipore), anti-FBL (1:2000; sc-374022, Santa Cruz Biotechnology), anti-H3K27me3 (1:1000; 31216, Active Motif), anti-Lamin A/C (1:2000; 4777S, Cell Signaling Technology), anti-Cleaved Caspase-3 (1:2000; 9661S, Cell Signaling Technology), anti-RPL7 (1:1000; 14583-1-AP, Proteintech), and anti-PCNA (1:2000, 10205-2-AP, Proteintech). β-actin (HRP-60008, Proteintech), anti-Vinculin (1:5000, sc-25336, Santa Cruz Biotechnology) or GAPDH (sc-47724, Santa Cruz Biotechnology) were used as a loading control. Secondary antibodies used in this study include goat anti-mouse IgG H&L (HRP) (ab6789, abcam) and goat anti-rabbit IgG H&L (HRP) (ab6721, abcam).

### Polysome profiling

Cells in 15-cm dish were treated with cycloheximide (CHX) (C4859, Sigma-Aldrich) at 100 μg/mL for 10 min. The medium was removed, and cells were washed with ice-cold PBS for 3 times containing 100 μg/ml CHX and harvested in 600 μl lysis buffer (20 mM HEPES, pH 7.6, 100 mM KCl, 5 mM MgCl_2_, 100 µg/ml CHX, 1% Triton X-100, 1% protease inhibitor cocktail and 40 U/ml RNase inhibitor). The samples were lysis on ice for 30 min. Then, the samples were centrifuged at 16,000 g for 15 min at 4 °C. Equal amounts of samples, as determined by absorbance at 260 nm, were layered on top of a 10% to 50% sucrose gradient (formed by the Gradient Master from BioComp Instruments) containing 20 mM HEPES pH 7.6, 100 mM KCl, 5 mM MgCl_2_, 100 μg /mL CHX, 1 × protease inhibitor and 20 U/mL RNase inhibitor (EO0382, Thermo Fisher Scientific). After centrifuged on an Optima L-100 XP Ultracentrifuge (rotor SW41i) at 41,000 rpm for 1.5 h at 4 °C, the samples were then fractionated into 20 fractions using the Piston Gradient Fractionator (BioComp Instruments) coupled with a fraction collector (Gilson) and a ECONO UV monitor (BioRad). The absorbance of 260 of each fraction was recorded. The fractions were then subjected to western blotting to evaluate the protein localization. RNA was purified from fractions using Trizol LS reagent (10-296-010, Thermo Fisher Scientific) and then subjected to RT-qPCR analysis to check the mRNA level of the genes.

### Proximity ligation assay (PLA)

HepG2 cells were seeded on an 8-well chamber slide (154534, Thermo Fisher Scientific). After three washes with PBS, the cells were fixed with 4% paraformaldehyde for 15 min at room temperature. Then, the cells were incubated with 1 × Permeabilization Buffer (00-8333-56, Thermo Fisher Scientific) for 15 min, followed by blocked with Duolink block solution for 1 h at room temperature and incubated overnight at 4 °C with the following antibodies: (1) mouse anti-Flag (F3165, Sigma-Aldrich) and rabbit anti-eIF3a (ab86146, Abcam); (2) rabbit anti-METTL16 (HPA020352, Sigma-Aldrich) and mouse anti-eIF3b (sc-137214, Santa Cruz Biotechnology). The next day, cells were washed twice with a large volume of PBS and incubated in PLA probes (DUO92002 and DUO92004, Sigma-Aldrich) for 1 h at 37 °C. Then, the cells were washed with 1 × Duolink for two times In Situ Wash Buffer A (DUO82049, Sigma-Aldrich) and incubated with ligation mix at 37 °C for 30 min. Subsequently, the cells were washed with 1 × Duolink In situ Wash Buffer A twice and incubated with amplification mix (DUO92008, Sigma-Aldrich) at 37 °C for 100 min. Finally, the cells were washed twice with 1 × Duolink in situ wash buffer B, washed once with 0.01 × Buffer B and mounted with Duolink in situ mounting medium with DAPI (DUO82040, Sigma-Aldrich). The pictures were captured under LSM 880 confocal microscope (Zeiss, Germany).

### Surface sensing of translation (SUnSET)

SUnSET was performed as previously described [34] to detect protein synthesis in HepG2 and Huh7 cells. Briefly, the cells were seeded in 24-well plate and incubated in DMEM medium supplemented with puromycin (1 μg/mL) for 30–60 min. Proteins were extracted from the cells and western blotting was conducted using anti-puromycin (MABE343, EMD Millipore).

### RNA tethering experiment

RNA tethering experiment was performed as previously described [25]. The λN peptide sequence (MDAQTRRRERRAEKQAQWKAAN) was fused to the C termini of METTL3 and METTL16, and all these sequences were inserted into the pmiRNA1 vector. The reporter plasmids, including pGL3-BoxB (300 ng), pGL3 control (300 ng), and pRL-TK (10 ng; E2241, Promega), and the effector plasmids (pmiRNA1-METTL16-λN, pmiRNA1-METTL3-λN, or pmiRNA1-EV, 300 ng for each) were transfected into HepG2 cells in 24-well plates. The relative luciferase activities (protein levels) were assessed 48 h following transfection using the Dual-luciferase reporter assay system (E1910, Promega). The F-Luc activity was normalized to that of Renilla luciferase (R-Luc). The RNA samples were collected, digested with DNase and subjected to qPCR to determine the mRNA expression levels of F-Luc and R-Luc. Finally, the F-Luc activity was normalized to R-Luc to evaluate the translation efficiency (protein/RNA). The normalized F-Luc activity in the pGL3 control was set to one.

### RNA extraction, bulk RNA-seq and cDNA synthesis

Total RNA was extracted from cells using Direct-zol™ RNA MiniPrep Kits (R2052, Zymo Research) and RNA Clean & Concentrator-5 Kit (R1015, Zymo Research) according to the manufacturer’s instructions. For bulk RNA-seq, RNA library preparation was conducted using KAPA Stranded mRNA-Seq Kit (Illumina Platforms) (Kapa Biosystems, Wilmington, USA) 10 cycles of PCR amplification and purified by AxyPrep Mag PCR Clean-up kit (Thermo Fisher Scientific). For reverse transcriptase, the reaction was performed with 100–1000 ng of total RNA or immunoprecipitated RNA samples using the QuantiTect Reverse Transcription kit (205314, QIAGEN) following the manufacturer’s instructions.

### Quantitative RT-PCR analysis and northern blotting

Quantitative real-time PCR (qPCR) was performed with Maxima SYBR Green qPCR Master Mix (2X) (FEPK0253, Thermo Fisher) using a QuantStudio (TM) 7Flex Real-Time PCR system (Applied Biosystem). Target gene expression levels were normalized by house-keeping gene *GAPDH* or *ACTB*. Northern Bolts was performed with NorthernMax™ Kit (AM1940, Invitrogen™) according to the manufacturer’s protocol. RNA was loaded on denaturing agarose gels and the biotin (Bio) labeled antisense probes of ITS-1 and ITS-2 as previously reported [35]. Used the same cell number as a loading control [36]. All the primers and probes used in qPCR analysis and Northern Blots are listed in Additional file 1: Table S1.

### RNA Immunoprecipitation (RIP) qPCR and sequence

The RIP experiment was performed according to the protocol from Abcam (https://www.abcam.com/epigenetics/rna-immunoprecipitation-rip-protocol) with some modifications. Briefly, Flag-tagged METTL16, eIF3a and eIF3b overexpression HepG2 cells (two 15-cm plates) were washed twice by ice-cold PBS and cross-linked at 254 nm (150 mJ/cm^2^), collected and lysed in 1 ml M-PER buffer (78,501, Thermo Fisher Scientific) with 100 U/ml RNase inhibitor and 1 × protease inhibitor on ice for 30 min and sonicated using a Bioruptor Pico at 4 °C with 30 s ON, 30 s OFF for 10 cycles. The lysate was collected by centrifugation at 13,000 g for 10 min at 4 °C and the protein concentration was determined using a Bio-Rad protein assay. One-tenth volume of the supernatant was kept as “input”. 5 μg anti-FLAG antibody (F3165, Sigma-Aldrich), eIF3a (ab86146, Abcam) or IgG was conjugated to 50 μl Protein A/G magnetic beads (88803, Thermo Fisher Scientific) with rotation at 4 °C for 4 h, followed by three washes with RIP buffer (150 mM KCl, 25 mM Tris (pH 7.4), 5 mM EDTA, 0.5 mM DTT, 0.5% NP40) and incubation with pre-cleared cell lysate at 4 °C overnight. The next day, the supernatant was kept as “ID (Immunodepletion)” and the beads were washed three times with RIP buffer, and resuspended in 80 μl PBS, followed by DNA digestion (DNase I, EN0521, Thermo Fisher Scientific at 37 °C for 30 min) and protein digestion (Proteinase K, EO0492, Thermo Fisher Scientific, for 55 °C for 1 h). Both the input and immunoprecipitated RNA were finally recovered by using RNA Clean & Concentrator kit (R1014, Zymo Research). For RIP-seq, the libraries were constructed using the KAPA Stranded mRNA-Seq Kit (Kapa Biosystems) after depletion of rRNA.

### Immunofluorescence assay (IFA)

1 × 10^4^ cells were seeded on 8-well chamber slides (Bioland Scientific) 16 h before staining. Cells were washed with PBS for three times and then fixed in 4% paraformaldehyde for 15 min at room temperature. The cells were then incubated with 100% methanol at -20 °C for 10 min and rinsed by PBS for 5 min. After that, cells were blocked in blocking solution (5% serum and 0.3% TritonTM X-100 with PBS) for 1 h at room temperature and incubated overnight at 4 °C with following primary antibodies: METTL16 (1:100; HPA020352, Sigma-Aldrich), METTL3 (1:100; ab195352, Abcam), METTL14 (1:100; HPA038002, Sigma-Aldrich), FBL (1:200; sc-374022, Santa Cruz Biotechnology), SC35 (1:200; ab11826, Abcam), NPM1 (1:200; CL594-60096, Proteintech), Flag (1:200; F3165, Sigma-Aldrich) and HA (1:100; 51064-2-AP, Proteintech). After 3 times washed with PBS, the corresponding fluorescence-labelled secondary antibodies (1:200 each; goat anti–rabbit/mouse IgG (H + L) Alexa Fluor 488, 555 or 647 (1:100; 4408S, 4409S, 4410S, 4412S, 4413S, Cell Signaling Technology) were applied to stain the cells and incubated for 1 h at room temperature. After washing three times with PBS, slides were mounted with In Situ Mounting Medium with DAPI (DUO82040, Sigma-Aldrich). The pictures were captured under Zeiss LSM 880 confocal microscope (Zeiss, Germany) or Nikon structured illumination microscopy (N-SIM, version AR5.11.00 64 bit, Tokyo, Japan) and analyzed by Zeiss ZEN, ImageJ and QuPath software.

### Cell fractionation

Nuclear RNA was isolated followed previous protocol [37] with some modifications. Briefly, 5 × 10^6^ Huh7 cells were collect with 1 ml ice-cold PBS/1 mM EDTA buffer. After that, the cell pellets were lysis by 200 μl ice cold lysis buffer (10 mM Tris–HCl, pH = 7.5, 0.05% NP40, 150 mM NaCl) incubated on ice for 5 min, then gently pipetted up the cell lysate over 2.5 volumes of chilled sucrose cushion (24% RNase-free sucrose in lysis buffer) and centrifuged at 4 °C with 15,000 g for 10 min. Supernatant was collected for cytoplasmic fraction and pellets for nuclei. RNA was purified from fractions using Trizol LS reagent (10–296-010, Thermo Fisher Scientific) and then subjected to Northern blotting and cDNA synthesis. The fractions were then subjected to western blotting to evaluate the isolation efficiency.

### Global proteomic analysis

HepG2 cells were washed twice with phosphate-buffered saline and lysed in 1 ml RIPA buffer (87787, Thermo Fisher Scientific) containing 1 × protease inhibitor cocktail (78438, Thermo Fisher Scientific) and 1 × phosphatase inhibitor cocktail (78426, Thermo Fisher Scientific) for 20 min on ice. The lysate was centrifuged at 13,000 g for 20 min, and protein concentration was measured by BCA method. After trypsin digestion, the peptides were labeled with TMT-6plex and were analyzed using Mass Spectrometer.

### Data analysis

For bulk RNA-seq, libraries were sequenced by an Illumina HiSeq 2500 (Illumina, San Diego, CA, USA) instrument in a 101 bp paired-end run, generated by the TruSeq SR Cluster Kit V4-cBot-HS (Illumina). After sequencing, reads were trimmed and masked for low-quality sequence by Cutadapt [38], and then mapped to the GRCh38 reference genome by STAR [39]. Per million mapped reads (RPKM) of each gene were calculated by RSEM [40].

For RIP-seq data, Samples were sequenced by NovaSeq 6000 platform. After sequencing, all reads were mapped to GRCh38 reference genome by STAR. The RNA–protein binding locations were determined using exomePeak [41] with default parameters. The potential targets were also identified using the normalized abundance of both the input and immunoprecipitated RNA, and enriched using MSigDB [42].

### Statistical analysis

Representative data from ≥ 3 independent experiments were analyzed with Graphpad Prism 9 and were presented as mean ± standard deviation (SD) or mean ± standard error of the mean (SEM). Statistical Significance was calculated using two-tailed, unpaired Student’s *t test*, paired *t test* and two-way ANOVA as indicated in the figure legends. Pearson tests were performed for correlation analysis. *P* values less than 0.05 were considered statistically significant. NS, not significant. For all violin plots, the median, 25th and 75th percentiles, minimum and maximum values are shown. Each sequence RNA sample has two biological replicates. For other experiments, the number of replicates is indicated in the figure legends. All Western blotting, Northern blotting and polysome profiling images are representatives of at least two independent experiments.

## Results

### Mettl16 is essential for de novo hepatocarcinogenesis, but not normal hepatogenesis

**Fig. 1:**
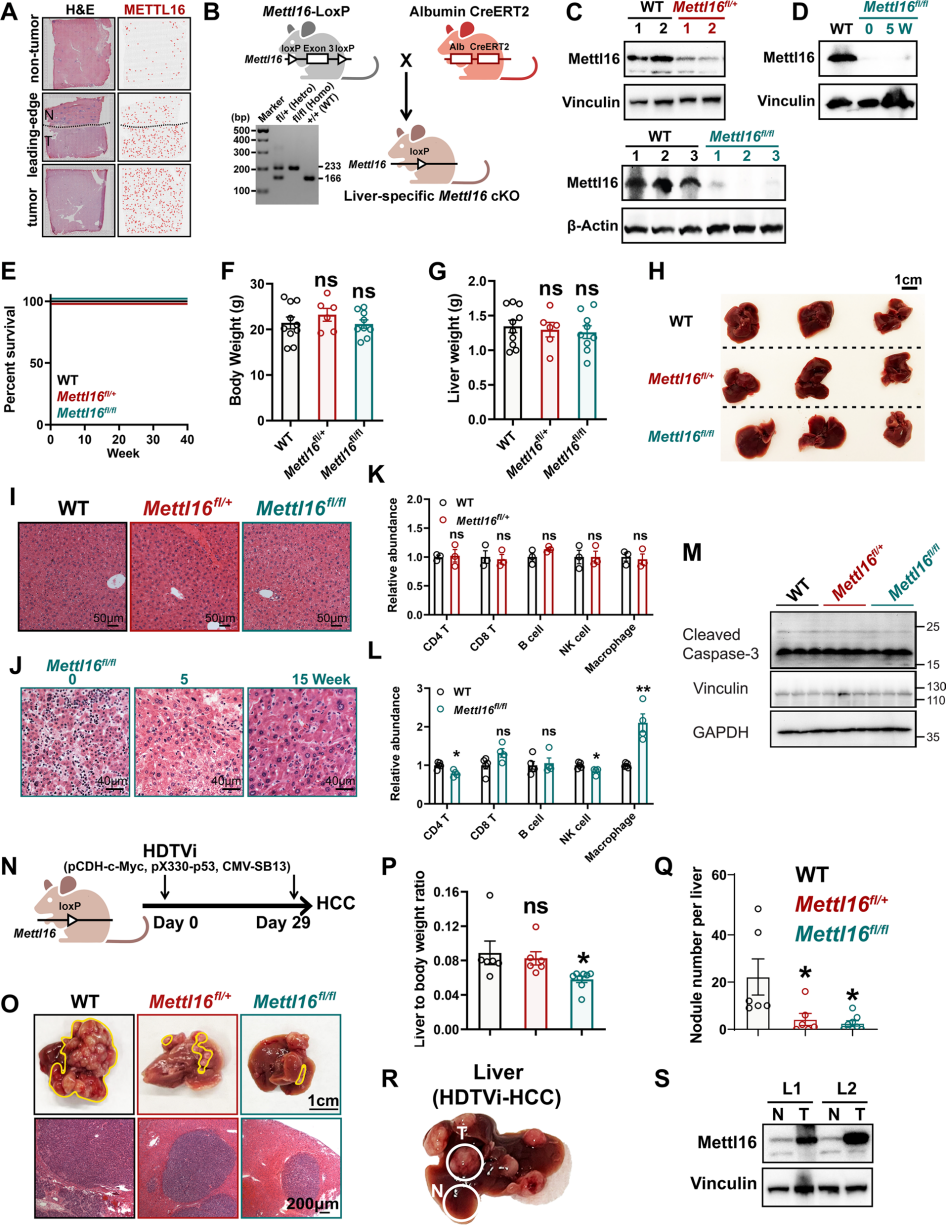
Mettl16 is essential for de novo hepatocarcinogenesis, but dispensable for normal hepatogenesis. **A** Spatial feature plots of METTL16 expression in HCC patient. N, non-tumor region; T, tumor region. **B** Schematic of the design and representative genotyping results of the liver-specific *Mettl16* conditional KO (cKO) mouse model. **C** Western blotting showing the *Mettl16* KO efficiency of liver of *Mettl16* wild-type (WT), heterozygous cKO (*Mettl16*^*fl/*+^), and homozygous cKO (*Mettl16*^*fl/fl*^) mice. **D** Western blotting showing the *Mettl16* KO efficiency in the livers of 0- and 5-week-old homozygous cKO mice. **E** Kaplan–Meier survival curves of *Mettl16* WT, heterozygous cKO and homozygous cKO mice. **F**, **G** Body (**F**) and liver (**G**) weight of adult *Mettl16* WT, heterozygous cKO and homozygous cKO mice (n = 10 for WT; n = 6 for *Mettl16*^*fl/+*^; n = 9 for *Mettl16*^*fl/fl*^; mean ± SEM). **H** Representative liver images of adult *Mettl16* WT, heterozygous cKO and homozygous cKO mice. **I** Representative H&E staining images of adult *Mettl16* WT, heterozygous cKO and homozygous cKO mice. **J** Representative H&E staining images of liver sections from *Mettl16* cKO mice at indicated time points postnatally. **K**, **L** Relative abundance of CD4^+ ^T cells, CD8^+ ^T cells, B cells, NK cells, and macrophages in *Mettl16* heterozygous cKO (**K**) and homozygous cKO (**L**) mice as compared to *Mettl16* WT (n = 3–5; mean ± SEM). **M** Western blotting showing the cleaved Caspase-3 level in the livers of *Mettl16* WT, heterozygous cKO, and homozygous cKO mice. **N** Schematic of HDTVi-induced de novo hepatocarcinogenesis model. **O** Representative liver (upper panel) and H&E staining (lower panel) images of tumors generated in *Mettl16* WT, heterozygous cKO, and homozygous cKO mice. **P**, **Q** The bar plots showing the ratio of liver weight to body weight (**P**) and the nodule numbers per liver (**Q**) (n = 6–8; mean ± SEM). **R** Representative images showing the tumor (T) and adjacent normal (N) tissue in the HDTVi-HCC model. **S** Western blotting showing the expression levels of *Mettl16* in HCC tumors and adjacent normal liver tissues. Statistical analyses: un-paired *t-test* (**F**, **G**, **K**, **L**, **P**, **Q**). ns, not significantly; *P < 0.05; **P < 0.01

Through spatial transcriptomic analysis of HCC patients [43], we observe high expression levels of METTL16, an enzyme responsible for RNA m^6^A modification, within HCC tumor regions compared to adjacent normal tissue or non-tumor regions (Fig. 1A). This finding implies that dysregulation of METTL16 might be involved in HCC pathogenesis. To evaluate the role of Mettl16 in HCC development and normal hepatogenesis, we created liver-specific *Mettl16* conditional KO (cKO) mice (*Mettl16*^*fl/fl*^-Alb-Cre) through crossing our recently generated *Mettl16*^*fl/fl*^ mice [44] with Albumin-Cre mice (Fig. 1B). Our Western blotting results verified that Alb-Cre led to a specific depletion of *Mettl16* in the liver, but not in other organs (Fig. 1C, D and Additional file 1: Fig. S1A). We then used *Mettl16*^*fl/fl*^-Alb-Cre mice to assess the role of Mettl16 in normal hepatogenesis and development. Neither heterozygous nor homozygous *Mettl16* KO showed a significant impact on the survival (Fig. 1E) or the body weight of the mice (Fig. 1F and Additional file 1: Fig. S1B). In addition, liver-specific *Mettl16* KO did not influence liver weight (Fig. 1G), liver size (Fig. 1H), or the weight of other critical organs, including heart, lung, spleen, pancreas, and kidney (Additional file 1: Fig. S1C). H&E staining showed that heterozygous or homozygous *Mettl16* KO had little impact on the structure of these organs (Fig. 1 I, J and Additional file 1: Fig. S1D). Considering that liver is the home to a diversity of immunologic cells and liver immunity plays a crucial role during HCC development [45–49], we also investigated whether liver-specific *Mettl16* KO could influence the populations of immune cells in liver via flow cytometry. Heterozygous *Mettl16* KO did not show any significant effect on the population of CD4^+^ T cells, CD8^+^ T cells, B cells, natural killer (NK) cells, and macrophages (Fig. 1K) in the liver. Homozygous *Mettl16* KO led to a moderate decrease of CD4^+^ T cells and NK cells, but a substantial increase of macrophages, with no influence on the population of CD8^+^ T cells and B cells in the liver (Fig. 1L and Additional file 1: Fig. S1E). Of note, neither heterozygous nor homozygous *Mettl16* KO showed profound effect on cleaved Caspase-3 level in livers, further validating that *Mettl16* KO had little effect on normal liver homeostasis (Fig. 1M).

To investigate the function of *Mettl16* in HCC initiation, we employed the hydrodynamic tail-vein injection (HDTVi)-induced de novo hepatocarcinogenesis model (Fig. 1N). Both heterozygous and especially homozygous *Mettl16* KO remarkably suppressed HCC development, as demonstrated by the reduction in HCC size and nodule number (Fig. 1O–Q). Similar to that shown in the normal mouse model (see Fig. 1F), heterozygous or homozygous *Mettl16* KO did not show any significant impact on body weight in the HDTVi-mediated HCC models either (Figure S1F-S1G). More interestingly, we collected the protein samples from HDTVi-HCC tumors and their adjacent normal liver tissues (Fig. 1R) and found that Mettl16 abundance is much higher in HDTVi-HCC tumors than normal liver tissues (Fig. 1S and Additional file 1: Fig. S1H), reiterating the tumor-promoting role of Mettl16 in de novo hepatocarcinogenesis. Taken together, *Mettl16* deletion has a moderate impact on normal hepatogenesis, but significantly suppresses HCC initiation and development.

### METTL16 potentiates CSC frequency/self-renewal and HCC progression

**Fig. 2:**
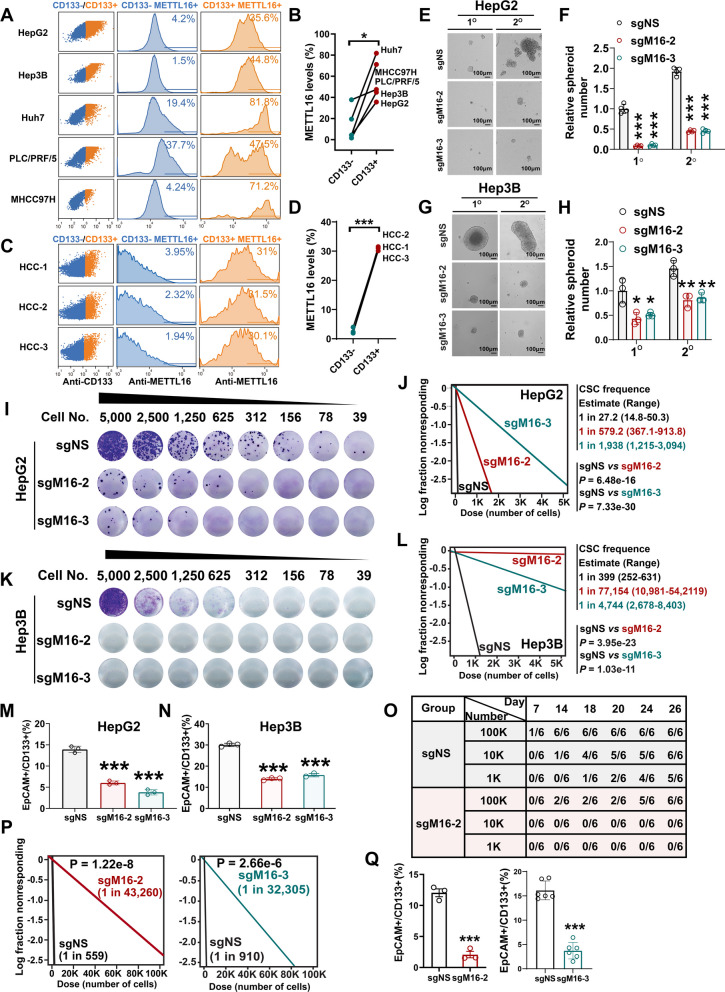
METTL16 is highly expressed in liver CSCs and genetic depletion of *METTL16* attenuates liver CSC self-renewal. **A**, **B** Histogram plot (**A**) and the statistical results (**B**) showing METTL16 abundance in CD133^−^ and CD133^+^ populations in HCC cell lines (n = 5). **C**, **D** Histogram plot (**C**) and the statistical results (**D**) showing METTL16 abundance in CD133^−^ and CD133^+^ populations in HCC tumors (n = 3). **E**, **F** Representative images (**E**) and the statistical results (**F**) showing the effects of *METTL16* KO on liver CSC maintenance as determined by spheroid formation assay in HepG2 cells (n = 4; mean ± SD). **G**, **H** Representative images (**G**) and the statistical results (**H**) showing the effects of *METTL16* KO on liver CSC maintenance as determined by spheroid formation assay in Hep3B cells (n = 3; mean ± SD). **I**, **J** Representative images (**I**) and the statistical results (**J**) showing the effects of *METTL16* KO on liver CSC frequency as determined by in vitro limiting dilution assay (LDA) in HepG2 cells. **K**, **L** Representative images (**K**) and the statistical results (**L**) showing the effects of *METTL16* KO on liver CSC frequency as determined by in vitro LDA in Hep3B cells. **M**, **N** Percentage of liver CSCs in HepG2 (**M**) and Hep3B (**N**) cells upon *METTL16* KO as determined by flow cytometry (n = 3; mean ± SD). **O** Table showing the injected cell numbers and the ratios of xenograft tumors implanted with HepG2 cells at the indicated number of days post transplantation. **P** Quantitative and statistical results showing the effects of *METTL16* KO induced by sg*METTL16*-2 (left panel) and sg*METTL16-3* (right panel) on liver CSC frequency as determined by in vivo LDA. **Q** Percentage of liver CSCs in xenograft tumors implanted with HepG2 cells with or without *METTL16* KO [n = 3 (left panel); n = 6 (right panel); mean ± SD]. Statistical analyses: paired *t* test (**B**, **D**); un-paired *t* test (**F**, **H**, **M**, **N**, **Q**); extreme limiting dilution analysis (ELDA) (https://bioinf.wehi.edu.au/software/elda/) (**J**, **L**, **P**). *P < 0.05, **P < 0.01, ***P < 0.001

There are accumulating evidence suggesting the existence of liver CSCs, a small population of HCC cells with self-renewal property [3]. The liver CSCs are responsible for HCC initiation, progression, metastasis, and chemo-resistance [50]. We first evaluated the expression levels of METTL16 in CD133^+^ HCC cells, which represent the well-characterized liver CSCs [51]. Via intracellular flow cytometry staining, we found that METTL16 level is significantly higher in CD133^+^ CSCs than in the CD133^−^ HCC cells (Fig. 2A, B). Consistently, METTL16 level is also significantly higher in the CD133^+^ CSCs of primary HCC tumors than in the CD133^−^ tumor cells (Fig. 2C, D). To further understand the role of METTL16 in liver CSC self-renewal, we incorporated two commonly used hepatic tumor cell lines [52], Hep3B, which harbors an integrated hepatitis B virus (HBV) genome, and HepG2, which lacks HBV integration, for functional studies. *METTL16* KO substantially suppressed the spheroid-forming capability in the 3D cultures of both HepG2 cells (Fig. 2E, F) and Hep3B cells (Fig. 2G, H). To quantitatively assess their effect on liver CSC frequency, we further conducted in vitro limiting dilution assays (LDA) and showed that *METTL16* KO dramatically decreased liver CSC frequency in HepG2 cells (Fig. 2I, J) and Hep3B cells (Fig. 2K, L). Of note, genetic depletion of *METTL16* also led to a significant decrease of liver CSC makers, CD133, and EpCAM (Fig. 2M, N). Finally, we performed in vivo LDA to further validate the effect of *METTL16* KO induced by 2 distinct sgRNAs on liver CSC self-renewal ability. As expected, *METTL16* KO suppressed HCC tumor formation and growth (Fig. 2O and Additional file 1: S2A–D), significantly decreased the liver CSC frequency (Fig. 2P) and attenuated the expression of liver CSC markers in vivo (Fig. 2Q).

Amongst the 29 human METTL family members, *METTL16* stands out as the most important gene for the survival of HCC cells (Additional file 1: Fig. S2E). Furthermore, METTL16 is overexpressed at both mRNA and protein levels in HCC patients (Additional file 1: Fig. S2F, G). We then assess the effect of *METTL16* depletion on the growth of HCC cell lines with different genetic backgrounds [52], including Hep3B (with HBV genome), Huh7 (with HCV genome), and HepG2 (without HBV or HCV). *METTL16* KO dramatically suppressed the growth of HCC cells (Additional file 1: Fig. S2H–K), which could be completely rescued by overexpression of wild-type (WT) METTL16 (sgRNA-resistant), demonstrating the high specificity of our sgRNA (Additional file 1: Fig. S2L–N). Moreover, *METTL16* KO-mediated growth inhibition could be also partially reverted by forced expression of catalytically inactive (PP185/186AA [25, 44]) METTL16 (sgRNA-resistant) (Additional file 1: Fig. S2L–N), suggesting both the methyltransferase-dependent and -independent functions of METTL16 contribute to its robust tumor-promoting role in HCC. Overall, our data highlights the crucial role of METTL16 in maintaining liver CSC maintenance and driving HCC progression, both in vitro and in vivo.

### eIF3a/b, the binding partners of METTL16, also promote liver CSC self-renewal and HCC progression

**Fig. 3:**
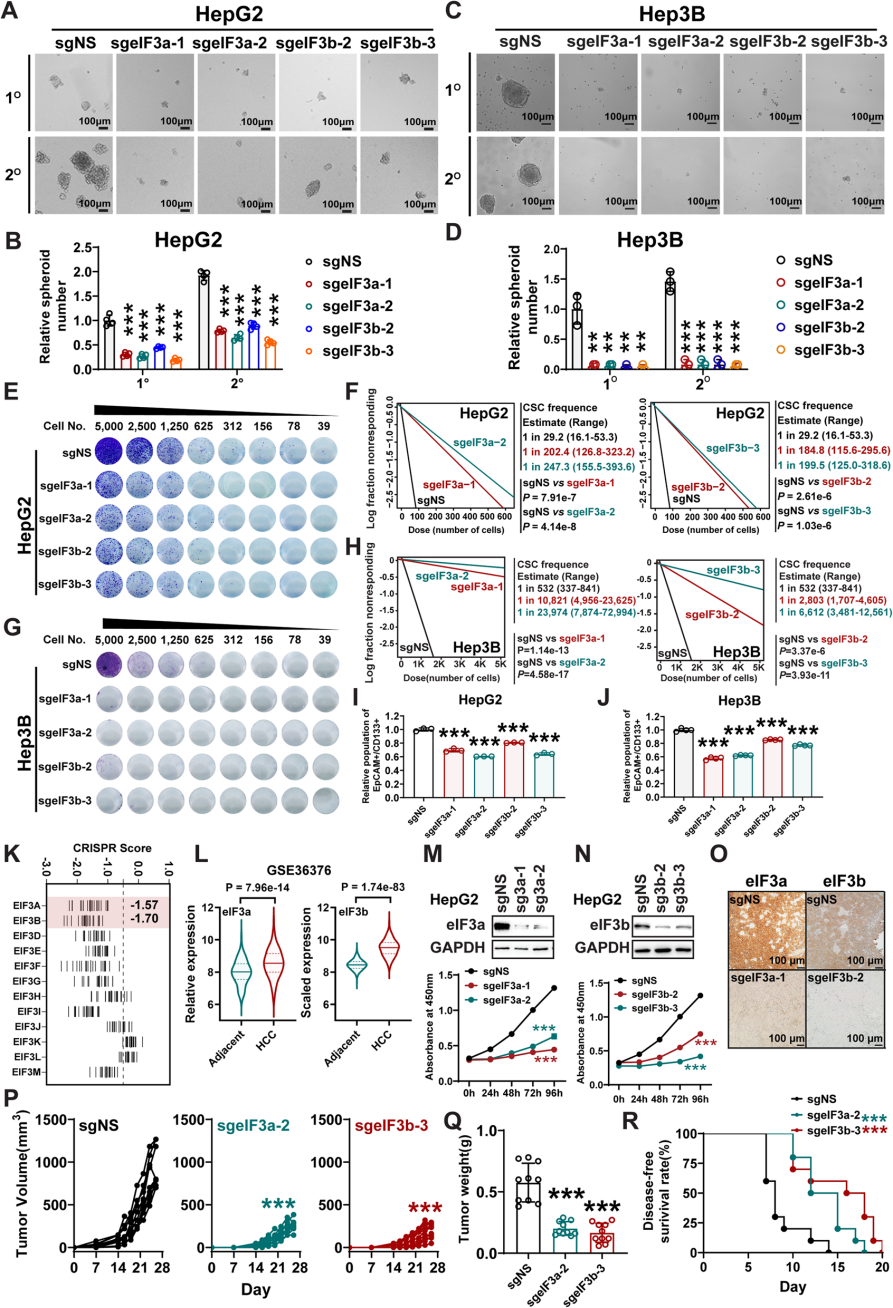
eIF3a and eIF3b, the binding partners of METTL16, promote CSC self-renewal and HCC development. **A**, **B** Representative images (**A**) and the statistical results (**B**) showing the effects of KO of *eIF3a* or *eIF3b* on liver CSC maintenance as determined by spheroid formation assay in HepG2 cells (n = 4; mean ± SD). **C**, **D** Representative images (**C**) and the statistical results (**D**) showing the effects of KO of *eIF3a* or *eIF3b* on liver CSC maintenance as determined by spheroid formation assay in Hep3B cells (n = 3; mean ± SD). **E**, **F** Representative images (**E**) and statistical results (**F**) showing the effects of KO of *eIF3a* or *eIF3b* on liver CSC self-renewal ability as determined by in vitro LDA in HepG2 cells. **G**, **H** Representative images (**G**) and statistical results (**H**) showing the effects of KO of *eIF3a* or *eIF3b* on liver CSC self-renewal ability as determined by in vitro LDA in Hep3B cells. **I**, **J** Effect of KO of *eIF3a* or *eIF3b* on the population of liver CSCs (EpCAM^+^/CD133^+^) in HepG2 (**I**) and Hep3B (**J**) cells upon as determined by flow cytometry [n = 3 (**I**), n = 4 (**J**); mean ± SD]. **K** CERES scores of eIF3 subunits from genome-scale CRISPR–Cas9 essentiality screens across 23 liver cancer cell lines. The raw data were downloaded from DepMap (https://depmap.org/portal/). The lower CERES score indicates a higher cancer dependency of the specific gene. Each stick represents one HCC cell line. **L** Comparison of the mRNA levels of *eIF3a* and *eIF3b* between human HCC tissues and normal controls. Adjacent = 193, HCC = 240. The three lines inside the violin plots are the first quartile, median and third quartile. **M**, **N** Effect of *eIF3a* (**M**) and *eIF3b* (**N**) KO on proliferation of HepG2 cells (n = 5; mean ± SD). **O** *eIF3a* or *eIF3b* KO efficacy in the xenograft liver tumors implanted with HepG2 cells as determined by immunohistochemistry. **P** Average growth curves of xenograft liver tumors upon KO of *eIF3a* or *eIF3b* (n = 10). **Q** Weights of the liver tumors on day 28 post injection (n = 10; mean ± SD). **R** Kaplan–Meier disease-free survival (DFS) of the xenograft models implanted with HepG2 cells with or without KO of *eIF3a* or *eIF3b* (n = 10). Statistical analyses: unpaired *t-test* (**B**, **D**, **I**, **J**, **L**, **Q**); Log-rank test (**R**); Two-way ANOVA (**M**, **N**, **P**). **P < 0.01, ***P < 0.001

Our previous study reported that METTL16 was associated with translation initiation machinery in cytosol to regulate mRNA translation initiation via interacting with eIF3a/b in HEK293T cells [25]. However, it is unclear about the biological function of eIF3a/b in HCC pathogenesis and CSC self-renewal. We showed that, analogous to *METTL16* KO, KO of *eIF3a* or *eIF3b* also remarkably suppressed the spheroid-forming capability of both HepG2 and Hep3B cell lines (Fig. 3A–D). Further in vitro LDA demonstrated that KO of *eIF3a* or *eIF3b* significantly decreased liver CSC frequency in various hepatic tumor cell lines with different genetic backgrounds, including HepG2, Hep3B, and Huh7 (Fig. 3E–H and Additional file 1: S3A–C). In consistency with those phenotypes, KO of *eIF3a/b* led to a significant decrease of CSC markers (Fig. 3I, J).

Amongst the multiprotein eIF3 complex, *eIF3a* and *eIF3b* are also the most essential genes for HCC cell growth (Fig. 3K). Furthermore, eIF3a and eIF3b are both overexpressed at mRNA and protein levels in HCC patients (Fig. 3L and S3D-S3E). KO of *eIF3a* or *eIF3b* also remarkably suppressed the growth of HepG2 (Fig. 3M, N), Huh7 (Additional file 1: Fig. S3F–G), and Hep3B (Additional file 1: Figs. S3H–I) in vitro. KO efficiency of *eIF3a* or *eIF3B* was validated by Western blotting (Additional file 1: Fig. S3J–K). Finally, we performed “human-in-mouse” xenotransplantation models with immunodeficient NSG mice to evaluate the function of eIF3a/b in HCC progression in vivo*.* KO of *eIF3a* or *eIF3b* significantly suppressed the growth of HCC tumors (Fig. 3O–Q and Additional file 1: Fig. S3L–O) and elongated the disease-free survival (DFS) of the recipient mice in vivo (Fig. 3R)*.* Overall, our in vitro and in vivo data demonstrated the crucial oncogenic roles of eIF3a/b in CSC frequency/self-renewal and HCC progression.

### METTL16-eIF3a/b interaction is responsible for enhanced mRNA translation and HCC cell proliferation

**Fig. 4:**
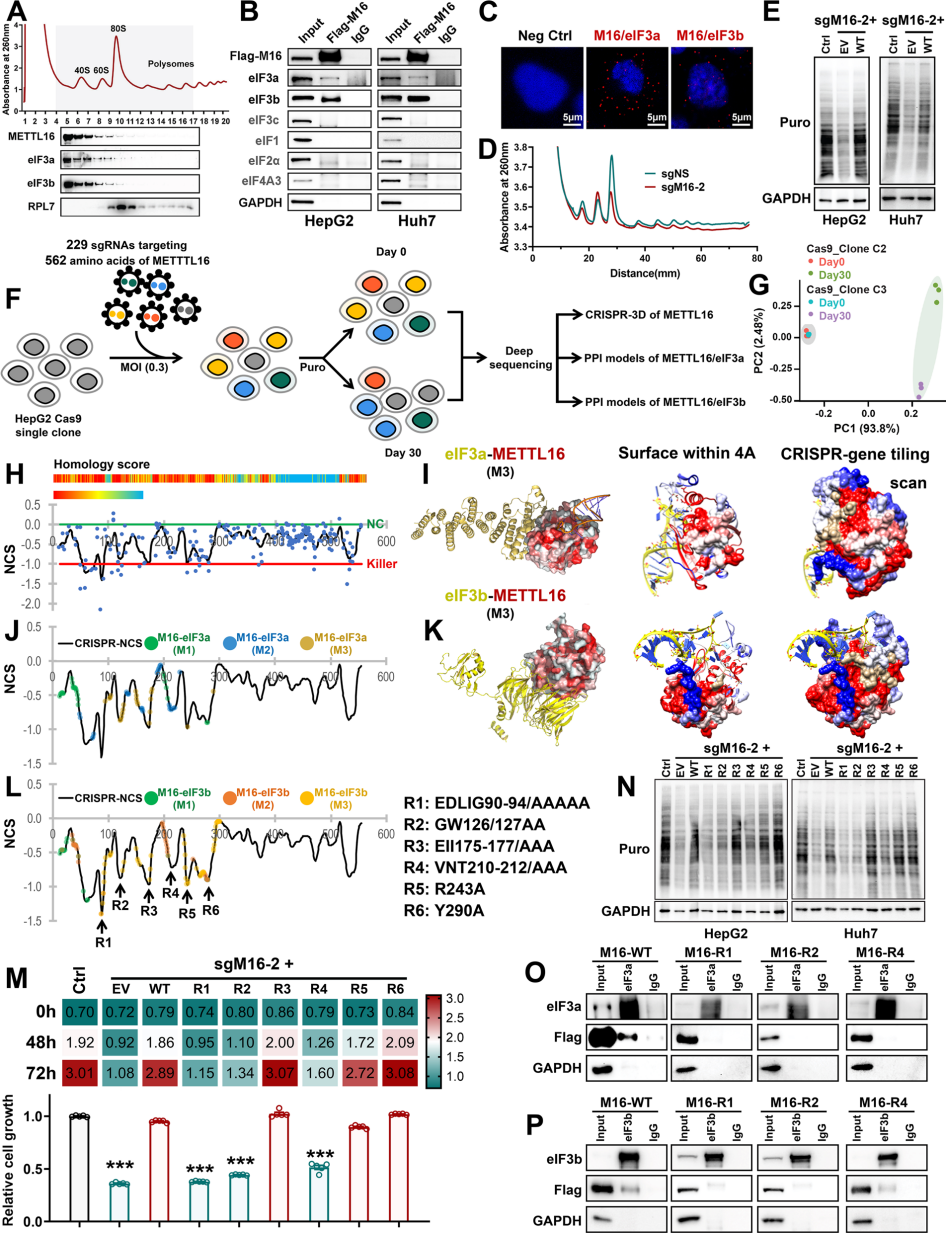
METTL16-eIF3a/b interactions are required for translation and proliferation promotion in HCC. **A** Polysome profiles of HepG2 cells as determined by sucrose density-gradient ultracentrifugation (top). The localization of eIF3a, eIF3b, METTL16, and RPL7 proteins were validated by Western blotting (bottom). **B** Representative Co-IP images showing the direct interaction between METTL16 and eIF members in HepG2 and Huh7 cells. **C** In situ detection of METTL16–eIF3a and METTL16–eIF3b interactions through PLA in HepG2 cells. **D** Representative polysome profiles of Huh7 cells upon *METTL16* KO. Data are representative of 3 independent experiments. **E** Representative Western blotting images of SUnSET assays used to quantify the amount of nascent [puromycin (Puro)-labeled] peptides in Huh7 and HepG2 cells with *METTL16* KO and rescued expression. **F** Schematic describing our in-house CRISPR screening with two HepG2 Cas9 single clones and protein–protein interaction (PPI) models. **G** Principal component analysis (PCA) of CRISPR screening data from 2 groups of HepG2 Cas9 single clones on day 0 and day 30. **H** Normalized CRISPR score (NCS) of each sgRNA construct (dot) and smoothed score (line) of the METTL16-tiling survival screen in HepG2 Cas9 single clones. Top, Peptide homology alignment of METTL16 across different species. **I** The PPI model between eIF3a and METTL16-CRISPR gene tiling scan. Left, PPI modeled structure (model 3, M3); Middle, Visualization of METTL16 surface area within 4 amino acids (4A) from the predicted eIF3a binding models; Right, CRISPR gene tiling scan plotting of METTL16 from the predicted eIF3a binding models. **J** The amino acids on the METTL16 predicted to be within 4A distance to eIF3a. **K** The PPI model between eIF3b and METTL16-CRISPR gene tiling scan. Left, PPI modeled structure (model 3, M3); Middle, Visualization of METTL16 surface area within 4A from the predicted eIF3b binding models; Right, CRISPR gene tiling scan plotting of METTL16 from the predicted eIF3b binding models. **L** The amino acids on the METTL16 predicted to be within 4A distance to eIF3b. Regions 1–6 (R1-R6) were derived from high-density CRISPR gene tiling scans of METTL16. **M** Rescue effect of regions 1–6 mutated METTL16s on *METTL16* KO-induced cell proliferation suppression in Huh7 cells (n = 5; mean ± SEM). **N** Representative Western blotting images of SUnSET assays in Huh7 and HepG2 cells with *METTL16* KO and rescued expression. **O**, **P** Representative Co-IP images showing the direct interaction between eIF3a (**O**) or eIF3b (**P**) and METTL16 with R1, R2 or R4 mutations in HepG2 cells. Statistical analyses: unpaired *t-test* (**M**); ***P < 0.001

To investigate whether METTL16-eIF3a/b axis is essential for HCC progression, we first investigated their occurrence and interaction in HCC cells. Here, we showed that METTL16 and eIF3a/b were mainly distributed in 40S, 60S, and 80S monosomes in HCC cells (Fig. 4A). Through Co-immunoprecipitations (Co-IP) assay, we demonstrated that METTL16 specifically interacted with eIF3a and eIF3b, but not other eIF factors in HCC cells (Fig. 4B). Using in situ proximity ligation assay (PLA), we confirmed the physical interactions between METTL16 and eIF3a/b in the cytosol of HCC cells (Fig. 4C). In addition, *METTL16* KO remarkably suppressed mRNA translation efficiency and the opposite was true when METTL16 was overexpressed (Fig. 4D, E and Additional file 1: Fig. S4A).

To identify the functionally essential amino acids and regions of METTL16 for its interaction with eIF3a/b and for HCC cell survival, we performed high-density CRISPR screen to generate the CRISPR gene tiling scan structure of METTL16 and predicted the protein–protein interaction (PPI) models between METTL16 and eIF3a/b accordingly. The CRISPR-Cas9-based tiling KO screen was performed based on the concept that the sgRNAs targeting essential regions exhibits a higher drop efficiency compared to those targeting nonessential regions [27, 53, 54]. This approach aimed to identify the functionally essential regions of METTL16. We cloned a pooled library containing 229 sgRNAs covering *METTL16* (average targeting density 7.4 bp per sgRNA), 40 negative control sgRNAs, and 22 killing control sgRNAs (Fig. 4F and Additional file 1: Table S1). The screen was performed in 2 HepG2 Cas9 single clones and the samples were collected on day 0 and day 30 post library transduction and puromycin selection (Fig. 4G and Additional file 1: Fig. S4B). Of note, the sgRNAs targeting N-terminal methyltransferase domain of METTL16 dropped out more remarkably than those targeting its C-terminus (Fig. 4H). In addition, we identified several regions playing more essential roles than the region which is responsible for single-stranded RNA (ssRNA) interaction and m^6^A methyltransferase activity (including amino acids P185, P186, and F187; Fig. 4H and Additional file 1: Fig. S4C), confirming that METTL16 may exert m^6^A-independent function. Based on the METTL16 CRISPR gene tiling scan structure and PPI models, we identified 6 regions (R1–R6) that might be essential for tumor cell survival and predicted to interact with eIF3a/b (Fig. 4I–L and Additional file 1: Fig. S4D–I). We then mutated the amino acids within these 6 regions and conducted a series of rescue assays in *METTL16* KO cells to determine which amino acids are indispensable for tumor cell survival/growth and mRNA translation. Using MTT assay and SUnSET assay, we showed that mutations in R1 (E90-G94), R2 (G126 and W127), and R4 (V210-T212) failed to rescue *METTL16* KO-mediated growth suppression (Fig. 4M) and mRNA translation inhibition (Fig. 4N and Additional file 1: Fig. S4J) in HepG2 and Huh7 cells. Moreover, the R1 and R2 of METTL16 are also important for its direct interaction with eIF3a/b (Fig. 4O, P). Collectively, METTL16 directly associates with eIF3a/b in the cytosol, and such interaction, mainly through METTL16 R1 and R2 regions, plays an important role in facilitating mRNA translation and promoting HCC cell survival/proliferation.

### METTL16 elevates translation-associated pathways and enhances *eIF3a* mRNA translation efficiency to promote CSC self-renewal

**Fig. 5:**
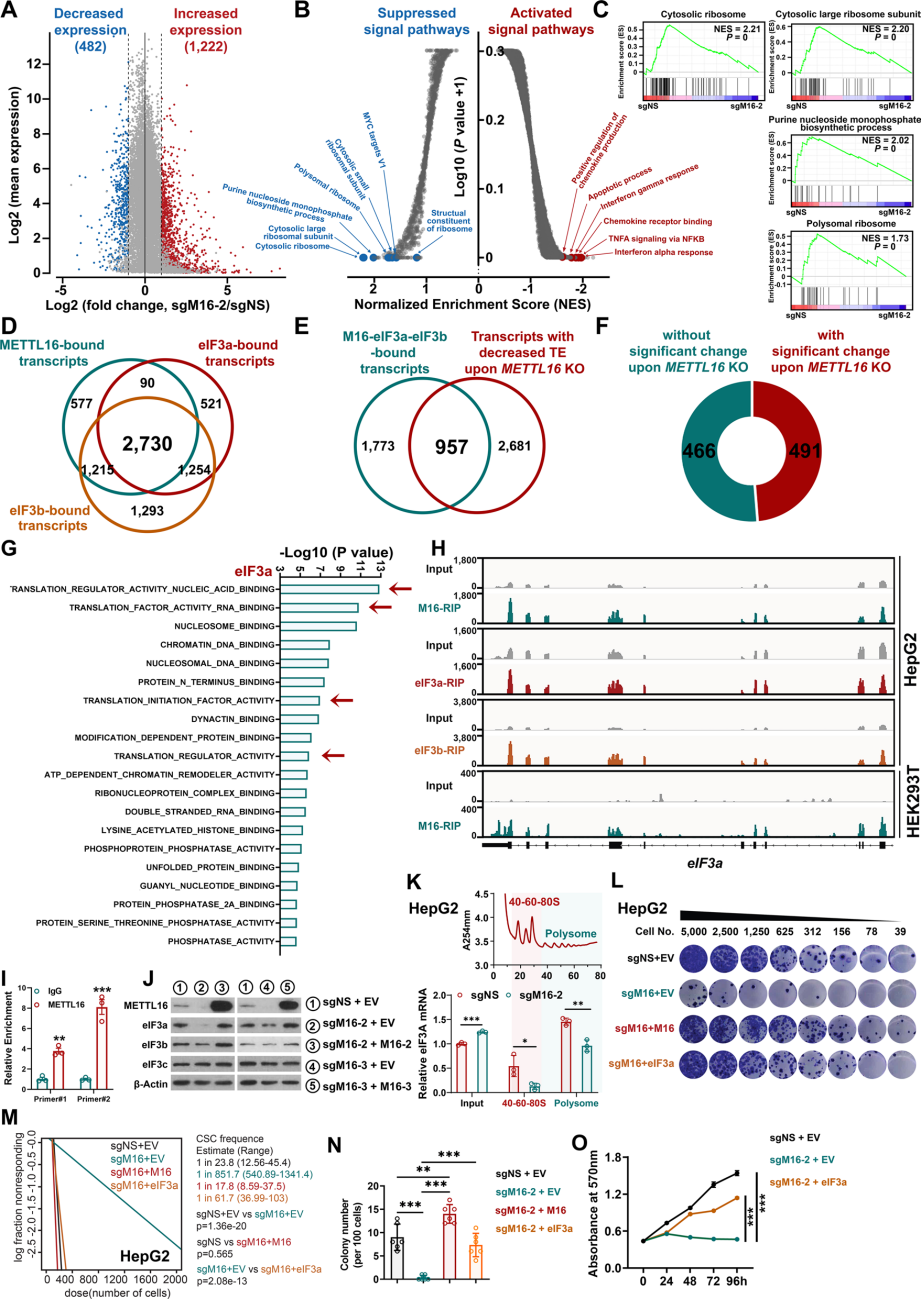
METTL16 enhances translation-associated pathways and *eIF3a* mRNA translation efficiency to promote CSC self-renewal. **A** MA plots displaying the decreased- and increased- expression genes in Huh7 cells upon *METTL16* KO. The dashed vertical lines represent Log_2_(fold change) = 1 or − 1. The significantly increased- or decreased- expression genes are shown in red and blue, respectively (*P* < 0.01); the grey dots indicate *P* ≥ 0.01. **B** Volcano plots showing the enriched gene signatures in Huh7 upon *METTL16* depletion. Here, we highlight the top decreased and increased pathways upon *METTL16* KO according to GSEA. **C** GSEA showing of top down-regulated gene signatures in Huh7 upon *METTL16* depletion. **D** Venn diagram showing the overlap between METTL16-, eIF3a-and eIF3b bound transcripts in HepG2 cells. **E** Venn diagram showing the overlap between the transcripts with decreased translation efficiency (TE) upon *METTL16* KO cells and the transcripts directly bound by METTL16-eIF3a- eIF3b. **F** Pie charts showing the distribution of 957 transcripts with or without significant mRNA level change upon *METTL16* KO. **G** The Gene Oncology Molecular Function (GO MF) enrichment analysis of the 466 transcripts without significant changes at mRNA levels upon *METTL16* KO. **H** Integrative genome viewer (IGV) browser tracks showing M16 (METTL16), eIF3a, and eIF3b binding peaks on *eIF3a* mRNA. The up three were conducted in HepG2 cells, while the bottom one was conducted in HEK293T cells. **I** METTL16 CLIP-qPCR analysis showing the interaction between METTL16 protein and *eIF3a* mRNA in HepG2 cells (n = 3; mean ± SEM). **J** Western blotting showing the expression levels of eIFs in HepG2 cells upon *METTL16* KO and rescue expression. **K** Ribo-qPCR showing the translation efficiency of *eIF3a* mRNA in HepG2 cells upon *METTL16* KO. **L**, **M** Representative images (**L**) and the statistical results (**M**) of liver CSC frequency as determined by in vitro LDA in HepG2 cells upon *METTL16* KO and rescue expression of METTL16 or eIF3a. **N** The bar plots showing the colony number in HepG2 cells upon *METTL16* KO and rescue expression of METTL16 or eIF3a (n = 6; mean ± SD). **O** The rescue effect of eIF3a on the cell proliferation of HepG2 cells upon *METTL16* KO (n = 3; mean ± SD). Statistical analyses: un-paired *t-test* (**I**, **K**, **N**); ELDA software (**M**); Two-way ANOVA (**O**). *P < 0.05, **P < 0.01, ***P < 0.001

To identify functionally essential downstream targets of METTL16 in HCC, we conducted transcriptome-wide RNA sequencing (RNA-seq), protein mass spectrometry (MS), RNA immunoprecipitation-sequencing (RIP-seq), and ribosome profiling. Analysis of RNA-seq data identified hundreds of differentially expressed genes (DEGs), including 482 significantly downregulated genes and 1,222 upregulated genes upon *METTL16 KO* in HCC cells (Fig. 5A and Additional file 1: Fig. S5A, B). Gene set enrichment analysis (GSEA) revealed that *METTL16* KO significantly activated apoptosis and chemokine response-related pathways (Fig. 5B and Additional file 1: Fig. S5C), while significantly suppressing translation-related pathways, purine nucleoside monophosphate biosynthetic process, and MYC targets (Fig. 5B, C and Additional file 1: Fig. S5D). Analysis of MS data identified 82 decreased proteins and 22 increased proteins in *METTL16* KO group (Additional file 1: Fig. S5E). This finding suggests that *METTL16* KO led to a global reduction in protein levels. Similarly, translation-related pathways were also significantly enriched in *METTL16* KO HCC cells (Additional file 1: Fig. S5F). This finding suggests that *METTL16* KO led to a global reduction in protein levels, which might be attributed to mRNA translation inhibition. Further via RIP-seq in HCC cells, we identified 4612, 4595, and 6492 directly binding transcripts of METTL16, eIF3a, and eIF3b, respectively (Additional file 1: Fig. S5G, H). Notably, majority of METTL16-bound transcripts (2730 out of 4612) could also be directly bound by eIF3a and eIF3b (Fig. 5D), suggesting that METTL16 and eIF3a/b regulate a large set of shared targets in HCC. Amongst the 2,730 transcripts bound by METTL16, eIF3a, and eIF3b, the translation efficiencies of 957 mRNA were significantly decreased when *METTL16* was depleted [25] (Fig. 5E). Amongst the 957 transcripts, we specifically focused on the 466 candidates whose mRNA levels were not significantly influenced by *METTL16* KO (Fig. 5F). Surprisingly, gene ontology (GO) analysis with the 466 candidates showed strong enrichment in the translation-associated pathways, including translation regulator activity, translation factor activity, and translation initiation factor (Fig. 5G). Of note, *eIF3a* participates in all these pathways. Strikingly, METTL16, eIF3a, and eIF3b directly bound *eIF3a* transcript, indicating *eIF3a* mRNA might be a translation-dependent target of METTL16 in HCC (Fig. 5 H, I and Additional file 1: Fig. S5I, J). Notably, our Western blotting data showed that *METTL16* KO dramatically inhibited protein expression of eIF3a, but not other translation initiation factors (Fig. 5J and Additional file 1: Fig. S5K); *METTL16* KO-mediated suppression of eIF3a could be completely reversed by ectopic expression of sgRNA-resistant METTL16 (Fig. 5J and Additional file 1: Fig. S5L). Our qPCR data didn’t reveal a significant decrease of *eIF3a* mRNA in HCC cells with *METTL16* KO and/or rescued expression (Additional file 1: Fig. S5M). Using polysome profiling, we showed that *METTL16* KO significantly decreased the translation efficiency of *eIF3a* transcript as *METTL16* KO led to a significant decrease of eIF3a transcript in monosomes and polysomes in HCC cells (Fig. 5K and Additional file 1: Fig. S5N, O).

Finally, to determine whether eIF3a down-regulation is necessary for the *METTL16 KO*-mediated HCC cell growth inhibition and CSC self-renewal suppression, we performed rescue assays via overexpression of eIF3a in *METTL16-*deficient HCC cells. Remarkably, forced expression of eIF3a could largely rescue the *METTL16* KO-induced inhibition on HCC cell growth and CSC self-renewal ability (Fig. 5L–O). Collectively, our results indicate that *eIF3a* is a translation-dependent and functionally essential target of METTL16 and highlight the profound effect of METTL16/eIF3a axis on mRNA translational control in HCC pathogenesis and CSC self-renewal.

### METTL16, but not METTL3 or METTL14, preferentially localizes to the nucleolus and facilitates ribosome biogenesis

**Fig. 6:**
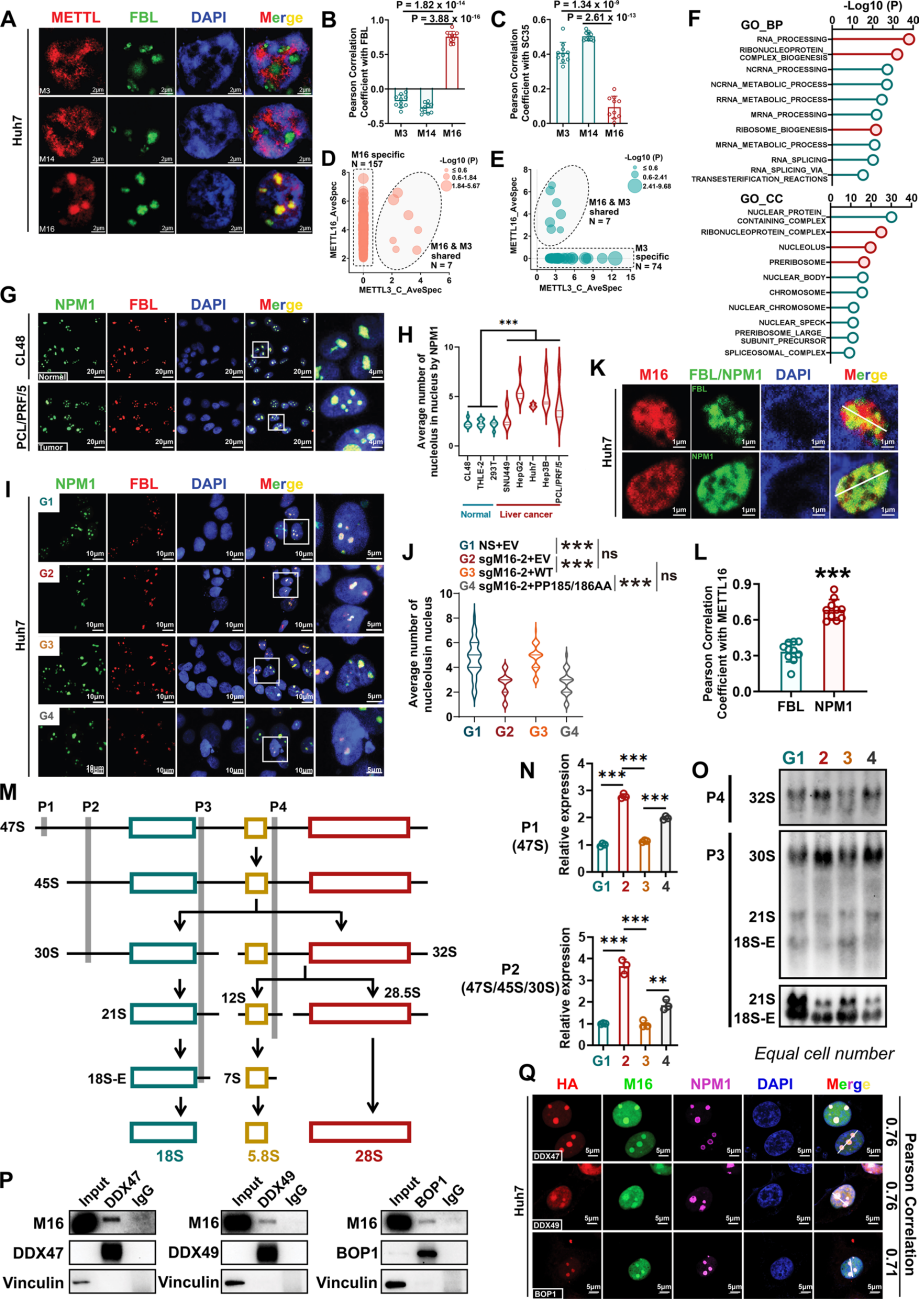
METTL16 preferentially localizes to the granular component (GC) of the nucleolus and facilitates rRNA processing and ribosome biogenesis. **A** Representative SIM images of FBL (green) and METTL members (red; including METTL3, M3; METTL14, M14; METTL16, M16) in the nucleus of Huh7 cells. FBL, nucleolar marker; DAPI, nuclear marker. **B**, **C** Pearson’s correlation analysis between distributions of FBL and the three METTL members (**B**) or between SC35 and the three METTL members in Huh7 cells (**C**) (n = 10; mean ± SD). SC35, nuclear speckle marker. **D**, **E** Bubble plots showing the METTL16-interacting (**D**) and METTL3-interacting (**E**) proteins identified by BioID assay. The size of each dot represents the *P* value of probability of binding of METTL16 or METTL3 with each protein. The proteins within the rectangle specifically interact with METTL16-BirA* (**D**) or METTL3-BirA* (**E**); while the proteins within the oval interact with both METTL16 and METTL3-BirA*. BirA*, BirA R118G variant. **F** GO enrichment analysis of the specific METTL16-interacting proteins. BP, biological process; CC, cellular component. **G** Representative confocal images showing the nucleolus in normal (CL48) and cancer (PCL/PRF/5) cells. **H** Statistical results of nucleolar numbers in normal cells and cancer cells (n > 40). **I**, **J** Representative confocal images (**I**) and the statistical results (**J**) showing the effects of *METTL16* KO and rescued expression on nucleolar numbers in Huh7 cells (n > 50). **K** Representative confocal images showing the subnucleolar localization of METTL16 in Huh7 cells. FBL, DFC marker; NPM1, GC marker. **L** Pearson’s correlation analysis between METTL16 and FBL or NPM1 in Huh7 cells (n = 10; mean ± SD). **M** Simplified schematic of rRNA processing and the probes we used for Northern blotting and qPCR. **N** The effects of *METTL16* KO and rescued expression on pre-rRNA levels in Huh7 cells as determined by qPCR (n = 3; mean ± SD). P1 was used to detect 47S pre-rRNA; while P2 was used to detect 47S, 45S, and 30S pre-rRNAs. **O** Representative images showing the effects of *METTL16* KO and rescued expression on pre-rRNA levels as determined by Northern blotting in HepG2 cells. **P** Representative Co-IP images showing the direct interaction between METTL16 and DDX47, DDX49, or BOP1 in HepG2 cells. **Q** Representative confocal images showing the colocalization of METTL16 with DDX47, DDX49, or BOP1 in the nucleolus of Huh7 cells. Statistical analyses: unpaired *t-test* (**B**, **C**, **H**, **J**, **L**, **N**); ns, not significant; **P < 0.01, ***P < 0.001

Given that loss-of-function mutation of METTL16 can partially reverse the growth suppression due to *METTL16* KO (see Additional file 1: Fig. S2L–N), we hypothesize that METTL16 might also exert methyltransferase-dependent function in HCC cells. According to current knowledge, m^6^A methyltransferases mainly exert their enzymatic activities in cell nucleus [55, 56]. We thus employed both structured illumination microscopy (SIM) and confocal imaging to define the exact subnuclear location of METTL16 in HCC cells. METTL3 and METTL14, the well-characterized mRNA m^6^A methyltransferases, were included as controls. In consistency with previous reports [55, 56], METTL3 and METTL14 were mainly localized in SC35-positive nuclear speckles (Additional file 1: Fig. S6A–D), which are sites for pre-mRNA processing and modifications [57]. In contrast, METTL16 was specifically enriched in the fibrillarin (FBL)-positive nucleolus instead of nuclear speckles (Fig. 6A–C and Additional file 1: Fig. S6E–M). Furthermore, we depleted the endogenous expression of METTL16 by CRISPR-Cas9 and then overexpressed sgRNA-resistant WT METTL16. We confirmed that both endogenous and exogenous METTL16 localized to the nucleolus (Additional file 1: Fig. S6N, O) *in cellulo*. As METTL16 and METTL3-METTL14 complex showed divergent subcellular localization, we speculated that they might have distinct binding partners. Indeed, we analyzed the *in-situ* binding proteins of METTL3 and METTL16, which were characterized by BioID assay [58], and identified that most (> 90%) of the proteins interacting with METTL16 do not associate with METTL3 (Fig. 6D, E and Additional file 1: Fig. S7A–C). Amongst the 164 in situ binding proteins of METTL16, only 7 may interact with METTL3 (Fig. 6D); similarly, only 7 out of the 81 binding proteins of METTL3 may interact with METTL16 (Fig. 6E). We then conducted Gene Ontology (GO) analysis with METTL16-specific binding partners and identified that these proteins are enriched in the pathways related to ribonucleoprotein complex biogenesis, ribosome biogenesis, nucleolus, and preribosome, which might be attributed to the specific enrichment of METTL16 within nucleolus (Fig. 6F).

To define whether the distribution of METTL16 in the nucleolus is related to HCC pathogenesis, we first compared nucleolar structural characteristics between non-malignant normal cells (CL-48, THLE-2, and HEK293T) and HCC cells (SUN449, HepG2, Huh7, Hep3B, and PCL/PRF/5). In contrast to normal cells, these HCC cells had significantly increased nucleolar numbers (Fig. 6G, H and Additional file 1: Fig. S7D–E). *METTL16* KO significantly decreased the nucleolar numbers in HCC cells, and this effect could be fully rescued by overexpression of WT METTL16, but not catalytically inactive METTL16 (PP185/186AA) (Fig. 6I, J and Additional file 1: Fig. S7F–G). The nucleolus is a highly dynamic structure composed of three subcompartments: fibrillar center (FC), dense fibrillar component (DFC) and granular component (GC) [59]. To determine how METTL16 maintains the nucleolar numbers, we further analyzed its subnucleolus localization and showed that METTL16 co-localized to the NPM1-positive GC compartment, but not to the FBL-positive DFC part (Fig. 6K, L and Additional file 1: Fig. S7H–K). Given that late rRNA processing predominantly takes place in the GC [60], we further examined the potential impact of METTL16 on rRNA processing. Our finding indicated that *METTL16* KO led to a robust accumulation of 47/45S, 32S, and 30S pre-ribosomal RNAs (pre-rRNAs), accompanied by a corresponding reduction of 21S and 18S-E rRNA levels in HCC cells (Fig. 6M–O and Additional file 1: Fig. S7L–O). Interestingly, expression of WT METTL16, but not METTL16-PP185/186AA, could fully rescue the rRNA processing blockage caused by *METTL16* KO (Fig. 6M–O), indicating that the catalytic activity of METTL16 is essential for pre-rRNA maturation.

Amongst all the METTL16-specific binding proteins revealed by the BioID assay, it’s noteworthy that DDX47 [61], DDX49 [62], and BOP1 [63] have been previously reported to have crucial roles in pre-rRNA processing. Through Co-IP assays, we confirmed the direct interactions between METTL16 and DDX47, DDX49, or BOP1 in HepG2 cells (Fig. 6P and Additional file 1: Fig. S7P). Subsequent immunofluorescence assay further validated the co-localization of METTL16 with DDX47, DDX49, or BOP1 in the nucleolus (Fig. 6Q and Additional file 1: Fig. S7Q-S7T). Furthermore, *METTL16* is significantly positively correlated with *DDX47*, *DDX49*, or *BOP1* in expression in The Cancer Genome Atlas Program (TCGA) Liver Hepatocellular Carcinoma (LIHC) database [64] (Additional file 1: Fig. S7U). In summary, our data conclusively demonstrate that the m^6^A methyltransferase METTL16 is specifically localized within the nucleolus, rather than nuclear speckles, and it plays a crucial role in modulating rRNA processing and ribosome biogenesis through interactions with DDX47, DDX49, and BOP1.

## Discussion

### Targeting METTL16 and mRNA translation as new antineoplastic avenues

Although considerable progress has been made in improving cancer survival, the field of cancer therapy still faces multiple key challenges in the pursuit of curing cancer, including relapse, metastasis, and drug resistance. The tumors rapidly evolve and adapt during therapy, partially due to the existence of CSCs. HCC accounts for over 80% of primary liver cancer cases and is the fourth most common cause of cancer-related death worldwide [65]. It remains difficult to treat because of the lack of effective therapeutic strategies and high rate of recurrence and heterogeneity. Liver CSCs are considered the master regulators of HCC initiation, progression, and tumor metastasis. However, how liver CSCs maintain their self-renewal property remains largely unknown. Using an integrative analysis of genome-wide CRISPR-Cas9 KO screening and TCGA-LIHC database, we reveal *METTL16* as the most essential genes for HCC survival. Genetic depletion of *METTL16* dramatically suppresses HCC initiation, progression, and liver CSC self-renewal via attenuating ribosome biogenesis and mRNA translation. While it has been reported that homozygous conventional *Mettl16* KO can potentially be embryonically lethal [66], liver-specific *Mettl16* cKO exhibits mild effects on normal hepatogenesis. This differs from Mettl3 and Mettl14 because liver-specific *Mettl3 cKO* and *Mettl14 cKO* cause severe liver injury or disruption of liver regeneration [67, 68]. Our findings indicate that METTL16 holds promise as a potential safe therapeutic target against HCC, while more studies are warranted to comprehensively evaluate its role in primary human hepatocytes.

The increases of nucleolar number and size as well as elevated ribosome biogenesis have profound effects on remodeling translational program and cancer plasticity, while the underlying mechanism has yet to be determined. We show the unexpected nucleolar localization of METTL16 facilitates rRNA processing and ribosome biogenesis in a methyltransferase-dependent manner, leading to increased nucleolar number in HCC cells. In addition, METTL16 extensively associates with translation initiation machinery via direct interaction with eIF3a/b to promote mRNA translation initiation. The direct interactions between METTL16 and eIF3a/b are essential for HCC growth and mRNA translation. Importantly, we have documented the PPI models between METTL16 and eIF3a/b and determined which functionally essential amino acids (regions) of METTL16 are necessary for the interplays, which provides structural information to develop specific inhibitor(s) to abolish the interaction for cancer treatment. We have also characterized *eIF3a* mRNA as a functionally essential and bona-fide downstream target of METTL16. This is substantiated by the fact that forced expression of eIF3a can largely rescue *METTL16* KO-mediated anti-tumor phenotypes, including inhibition of tumor growth and liver CSC self-renewal. Overall, our studies indicate that the oncogenic function of METTL16 relies on its role in reprogramming mRNA translation in cancer (e.g., HCC) cells, and highlight the therapeutic potential of targeting the METTL16/eIF3a axis and mRNA translation program for cancer treatment.

### METTL16 functions as a key player in protein synthesis and CSC stemness

Using the high-density CRISPR gene body scan which enables the identification of functional elements within a given protein by saturation mutagenesis achieved through CRISPR-mediated gene editing, we identify the methyltransferase activity-independent and functionally essential regions of METTL16 protein. This finding emphasizes that both the enzyme-dependent and -independent mechanisms are responsible for the robust oncogenic role of METTL16 in HCC cells [25]. Besides the nucleolar localization, METTL16 also directly associates with eIF3a/b, but not other eIFs, in the cytosol to enhance mRNA translation in cancer cells. The mutations of amino acids in two regions (90–94a.a. and 126/127a.a.) within the α-helix structure of METTL16 severely impair HCC cell survival/proliferation and mRNA translation and substantially disrupt the interaction between METTL16 and eIF3a/b. Despite multiple members in human METTL family have been characterized as m^6^A methyltransferases [24, 56, 69], METTL16 is the most essential one for the survival of cancer cells, which might be attributed to its methyltransferase activity-dependent and -independent functions and especially its pivotal role in protein synthesis, including rRNA processing, ribosome biogenesis and translation initiation.

Translational control of mRNA plays a central role in reshaping the plasticity of cancer cells to adapt to the hostile microenvironment and promote tumor progression and metastasis [7]. Such plasticity also endows cancer cells with the ability to dynamically transit between a differentiated state and an undifferentiated stem-like state [8]. Our results show that METTL16-induced translational promotion is important in the acquisition and maintenance of CSC stemness. Additionally, eIF3a/b, the translation-related binding partners of METTL16, also have a strong impact on CSC self-renewal and HCC progression. This finding indicates that METTL16-mediated translation initiation likely reprograms translational control in HCC to maintain tumor plasticity and CSC stemness.

### The distinct locations determine distinctive functions of m^6^A methyltransferases

As the most abundant modification in mRNAs, m^6^A functions at almost every stage of mRNA metabolism. The mRNA m^6^A modifications are mainly catalyzed by the METTL3-METTL14 complex, whose targets share a consensus sequence of DRACH with no obvious structural preferences. However, unlike the METTL3-METTL14 complex, METTL16 selectively methylates the structured RNAs where the critical m^6^A is present in a bulge and seems to have a distinct set of targets for m^6^A modification, including *MAT2A* mRNA and U6 snRNA [66]. In line with previous reports [55, 56], we show that METTL3-METTL14 predominantly resides in the nuclear speckles, the site for pre-mRNA processing and alternative splicing. In contrast, METTL16 is excluded from nuclear speckle, while accumulated in the nucleolus of HCC cells. Analysis of the BioID assay data showed that METTL16 and METTL3 have very different partner proteins. These findings suggest that, although METTL16 and METTL3-METTL14 are both recognized as m^6^A methyltransferases, they may have distinct RNA substrates and biological functions, owing to the different subcellular localizations.

The nucleolus is a phase-separated cell condensate and consists of three morphological subcompartments. The FC, at the core, is surrounded by the DFC, and both are embedded within liquid-like GC. Our data suggests that METTL16 colocalizes to the GC sub-nucleolar region via direct interaction with DDX47, DDX49, and BOP1, contributing to late-stage pre-rRNA maturation. Depletion of *METTL16* leads to a decrease of 21S and 18S-E rRNAs, associated with a corresponding increase of their precursors, 30S, 32S, 45S, and 47S rRNAs. *METTL16* KO-mediated inhibition of pre-rRNA processing results in a significant decrease of nucleolar numbers in tumor cells. More interesting, those effects can be fully reversed by overexpression of WT METTL16, but not catalytically inactive METTL16, demonstrating that METTL16’s methyltransferase activity is essential for its role in rRNA maturation and ribosome biogenesis. Previous studies from us and others have reported that METTL16 interacts with rRNAs [25, 70]. In consistent with our result, YbiN, the E. coli homolog of METTL16, is responsible for methylation of rRNA [71]. Therefore, we postulate that METTL16 directly methylates rRNAs, especially 47S pre-rRNA and/or its processing products, to guide rRNA processing and ribosome biogenesis. Nevertheless, further investigations are warranted to test this hypothesis. Furthermore, small nucleolar RNAs (snoRNAs) are also abundant in the nucleolus and essential for rRNA processing and modification [72]. snoRNAs are characterized as a new class of m^6^A-modified ncRNAs and have common secondary structures with internal bulges [73]. It would be also very intriguing to explore whether METTL16 is responsible for depositing RNA methylations on snoRNAs.

### Supplementary Information

**Additional file 1**. Supplementary tables and Figures.

## Data Availability

The RNA-seq data supporting the conclusions of this article is available in the gene expression omnibus (GEO) and made accessible under accession number GSE224008 (https://www.ncbi.nlm.nih.gov/geo/query/acc.cgi?acc=GSE224008). The RIP-seq data supporting the conclusions of this article is available in the GEO and made accessible under the accession number in GSE224009 (https://www.ncbi.nlm.nih.gov/geo/query/acc.cgi?acc=GSE224009).
