# Supplementary material for: METTL16 promotes liver cancer stem cell self-renewal via controlling ribosome biogenesis and mRNA translation
Source: J Hematol Oncol. 2024 Feb 1;17:7. doi: 10.1186/s13045-024-01526-9 (PMC10835888; doi:10.1186/s13045-024-01526-9)
Supplement: Supplementary file 1 — Additional file 1. Supplementary tables and Figures. [file 13045_2024_1526_MOESM1_ESM.docx]

Supplementary Data File for:

**METTL16 promotes liver cancer stem cell self-renewal via controlling ribosome biogenesis and mRNA translation**

Meilin Xue^1,4,16^, Lei Dong^1,14,16^, Honghai Zhang^1,16^, Yangchan Li^1,8^, Kangqiang Qiu^7^, Zhicong Zhao^1,9^, Min Gao^1^, Li Han^1,10^, Anthony K. N. Chan^1^, Wei Li^1^, Keith Leung^1^, Kitty Wang^1^, Sheela Pangeni Pokharel^1^, Ying Qing^1^, Wei Liu^1^, Xueer Wang^1^, Lili Ren^1^, Hongjie Bi^1^, Lu Yang^1^, Chao Shen^1^, Zhenhua Chen^1^, Laleh Melstrom^11^, Hongzhi Li^12^, Nikolai Timchenko^13^, Xiaolan Deng^1^, Wendong Huang^5,6^, Steven T. Rosen^2^, Jingyan Tian^15^, Lin Xu^14^, Jiajie Diao^7^, Chun-Wei Chen^1,2^, Jianjun Chen^1,2,3^ , Baiyong Shen^4,*^ Hao Chen^4,*^, Rui Su^1,2,*^

**Table of contents**

[**Supplementary Figure 1**](#_Toc129672294) 2

[**Supplementary Figure 2** 4](#_Toc129672295)

[**Supplementary Figure 3** 7](#_Toc129672296)

[**Supplementary Figure 4** 9](#_Toc129672297)

[**Supplementary Figure 5** 11](#_Toc129672298)

[**Supplementary Figure 6** 14](#_Toc129672299)

[**Supplementary Figure 7** 16](#_Toc129672300)

[**Supplementary Table 1. CRISPR screening library sequences** 20](#_Toc129672301)

[**Supplementary Table 2. List of oligonucleotides**](#_Toc129672302) 32

Supplementary Figures and Figure Legends

**Supplementary Figure 1**

**
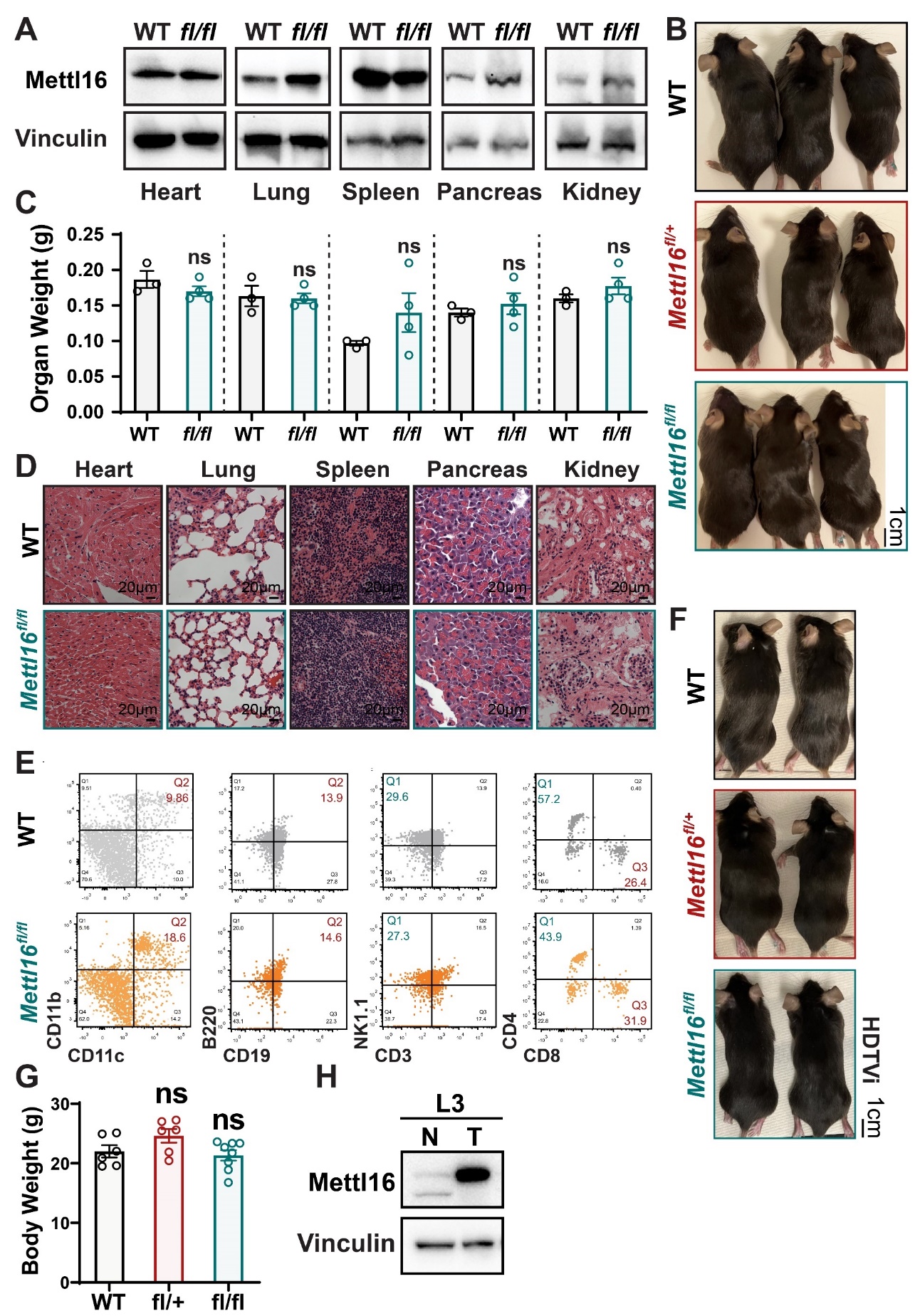
**

**Supplementary Figure 1. Liver-specific depletion of *Mettl16* is dispensable for normal hepatogenesis but attenuates hepatocarcinogenesis.**

(A) Western blotting showing the expression levels of *Mettl16* in the heart, lung, spleen, pancreas, and kidney of *Mettl16* wild-type (WT) and homozygous liver-specific cKO (*Mettl16^fl/fl^*) mice.

(B) Representative appearance of sex-matched WT, heterozygous *Mettl16* cKO, and homozygous *Mettl16* cKO mice at 5 weeks after birth.

(C) The bar plots showing the weight of the heart, lung, spleen, pancreas, and kidney in WT and homozygous *Mettl16* cKO mice.

(D) Representative H&E staining photographs of heart, lung, spleen, pancreas, and kidney of WT and homozygous *Mettl16* cKO mice.

(E) Representative FACS plots showing macrophages, B cells, NK cells, and T cells percentage in WT and homozygous *Mettl16* cKO mice.

(F) Representative appearance of sex-matched WT, heterozygous *Mettl16* cKO, and homozygous *Mettl16* cKO mice in HDTVi-HCC model.

(G) Body weight of WT, heterozygous *Mettl16* cKO, and homozygous *Mettl16* cKO mice (n = 8; mean ± SEM).

(H) Western blotting showing the expression levels of Mettl16 in HCC tumor and adjacent normal liver tissue. Vinculin was used as a loading control.

Statistical analyses: un-paired *t-test* (C, G); ns, not significant.

# **Supplementary Figure 2**

**
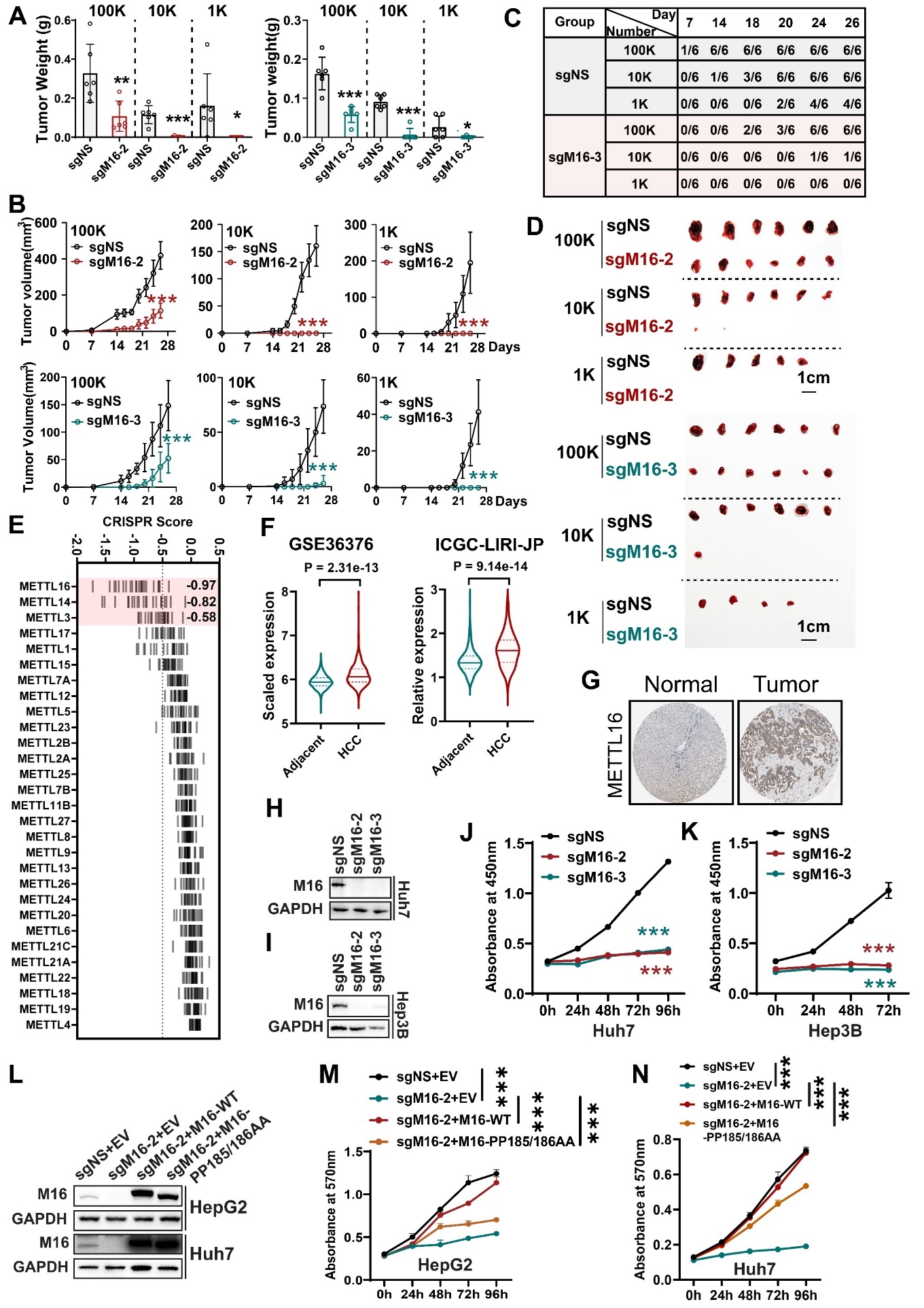
**

**Supplementary Figure 2. Genetic depletion of *METTL16* attenuates self-renewal/maintenance of liver CSCs and HCC progression.**

(A) Effect of *METTL16* KO in HepG2 cells induced by sg*METTL16*-2 (sgM16-2; left panel) and sg*METTL16*-3 (sgM16-3; right panel) on the weight of xenograft tumors at the endpoints (n = 6; mean ± SD).

(B) Average growth curves of xenograft tumors implanted with HepG2 cells with or without *METTL16* KO (n = 6; mean ± SEM).

(C) Table showing the implanted HepG2 cell numbers and the ratios of xenograft tumors at the indicated time points post transplantation.

(D) Tumor images at the endpoint of the xenograft models in the *in vivo* LDA.

(E) CERES scores of METTL family members from genome-scale CRISPR–Cas9 essentiality screens across 23 liver cancer cell lines. The raw data were downloaded from DepMap (<https://depmap.org/portal/>). As the CERES scores, 0 and -0.5 represent the median effects of nonessential genes and common core essential genes, respectively. The lower CERES score indicates a higher cancer dependency of the specific gene. Each stick represents an HCC cell line.

(F) Comparison of the mRNA levels of *METTL16* between human HCC tissues and normal controls. Left panel: adjacent = 193, HCC = 240; Right panel: adjacent = 177, HCC = 212. The three lines inside the violin plots are the first quartile, median and third quartile.

(G) Comparison of the METTL16 protein levels between human HCCs and normal controls. Representative panels were downloaded from The Human Protein Atlas (www.proteinatlas.org).

(H-I) Representative Western blotting images showing the *METTL16* KO efficiency in Huh7 (H) and Hep3B (I) cells.

(J-K) The effect of *METTL16* KO on the cell proliferation of Huh7 (J) and Hep3B (K) cells.

(L) Representative Western blotting images showing the *METTL16* KO and rescued expression efficiency in HepG2 and Huh7 cells.

(M-N) The effects of *METTL16* KO and rescued expression on the proliferation of HepG2 (M) and Huh7 (N) cells (n = 5; mean ± SD).

Statistical analyses: un-paired *t-test* (A, F), Two-way ANOVA (B, J, K, M, N); **P* < 0.05, ***P* < 0.01, ****P*<0.001.

# **Supplementary Figure 3**

# **
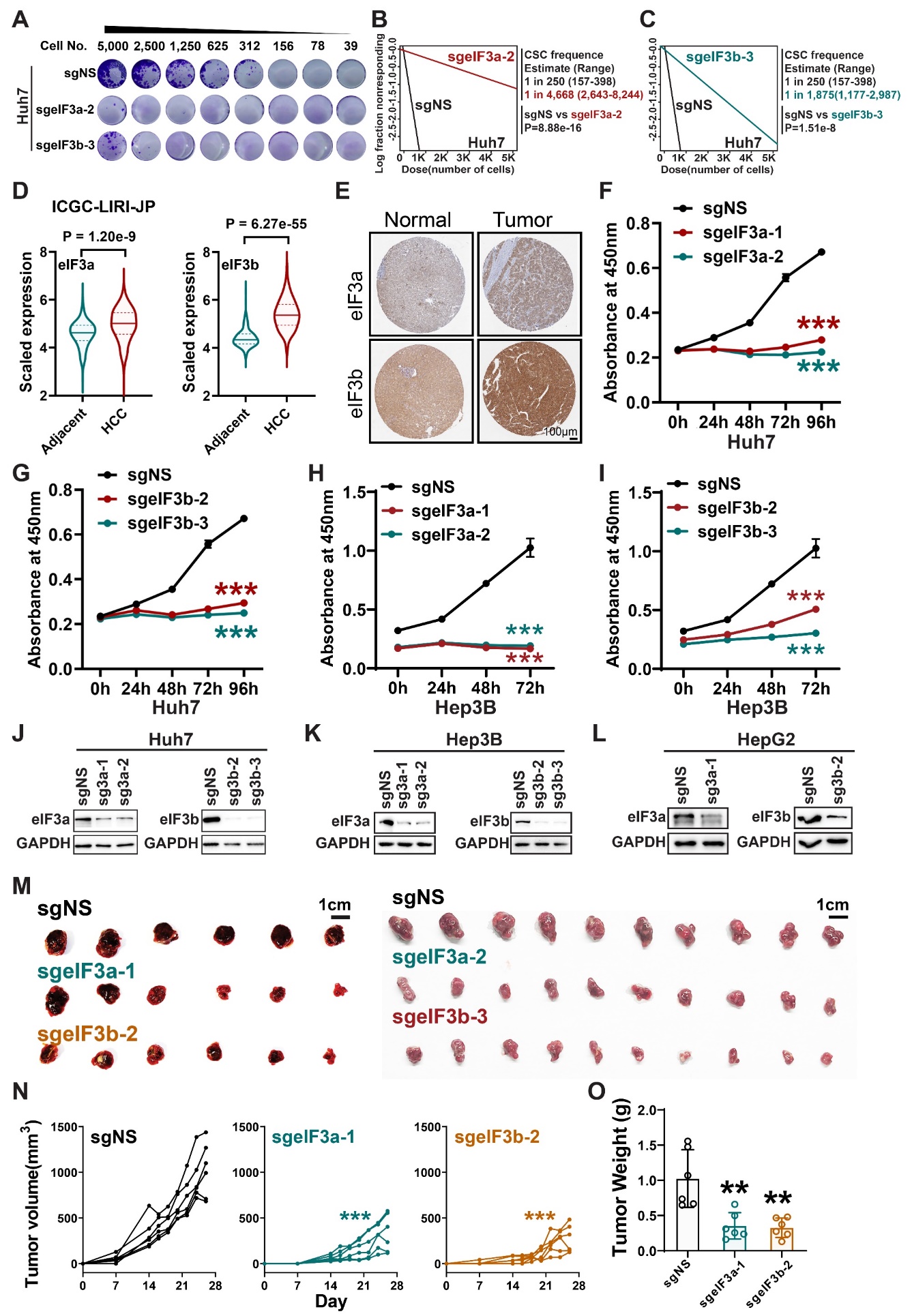
**

**Supplementary Figure 3. eIF3a and eIF3b are highly expressed in HCC, and genetic depletion of *eIF3a* or *eIF3b* attenuates liver CSC self-renewal and HCC progression.**

(A-C) Representative images (A) and statistical results (B-C) showing the effects of KO of *eIF3a* or *eIF3b* on liver CSC self-renewal ability as determined by *in vitro* LDA in Huh7 cells.

(D) Comparison of the mRNA levels of *eIF3a* and *eIF3b* between human HCC tissues and normal controls. Adjacent = 177, HCC = 212. The three lines inside the violin plots are the first quartile, median and third quartile.

(E) Comparison of the eIF3a and eIF3b protein levels between human HCCs and normal controls. Representative panels were downloaded from The Human Protein Atlas ([www.proteinatlas.org](http://www.proteinatlas.org)).

(F-G) The effect of KO of *eIF3a* (F) or *eIF3b* (G) on the cell proliferation of Huh7 cells.

(H-I) The effect of KO of *eIF3a* (H) or *eIF3b* (I) on the cell proliferation of Hep3B cells.

(J-K) Representative Western blotting images showing the KO efficiency of *eIF3a* or *eIF3b* in Huh7 (J) and Hep3B (K) cells.

(L) Western blotting showing the KO efficiency of *eIF3a* or *eIF3b* in HepG2 Cas9 single clone used in the xenograft models.

(M) Tumor images at the endpoint of the xenograft models implanted with HepG2 upon KO of *eIF3a* or *eIF3b*.

(N) Average growth curves of xenograft liver tumors upon KO of *eIF3a* or *eIF3b* (n = 6).

(O) Weights of the liver tumors on day 28 post injection (n = 6; mean ± SD).

Statistical analyses: ELDA (<https://bioinf.wehi.edu.au/software/elda/>) (B, C), un-paired *t-test* (D, O), Two-way ANOVA (F, G, H, I, N); ***P* < 0.01, ****P*<0.001.

# **Supplementary Figure 4**

**
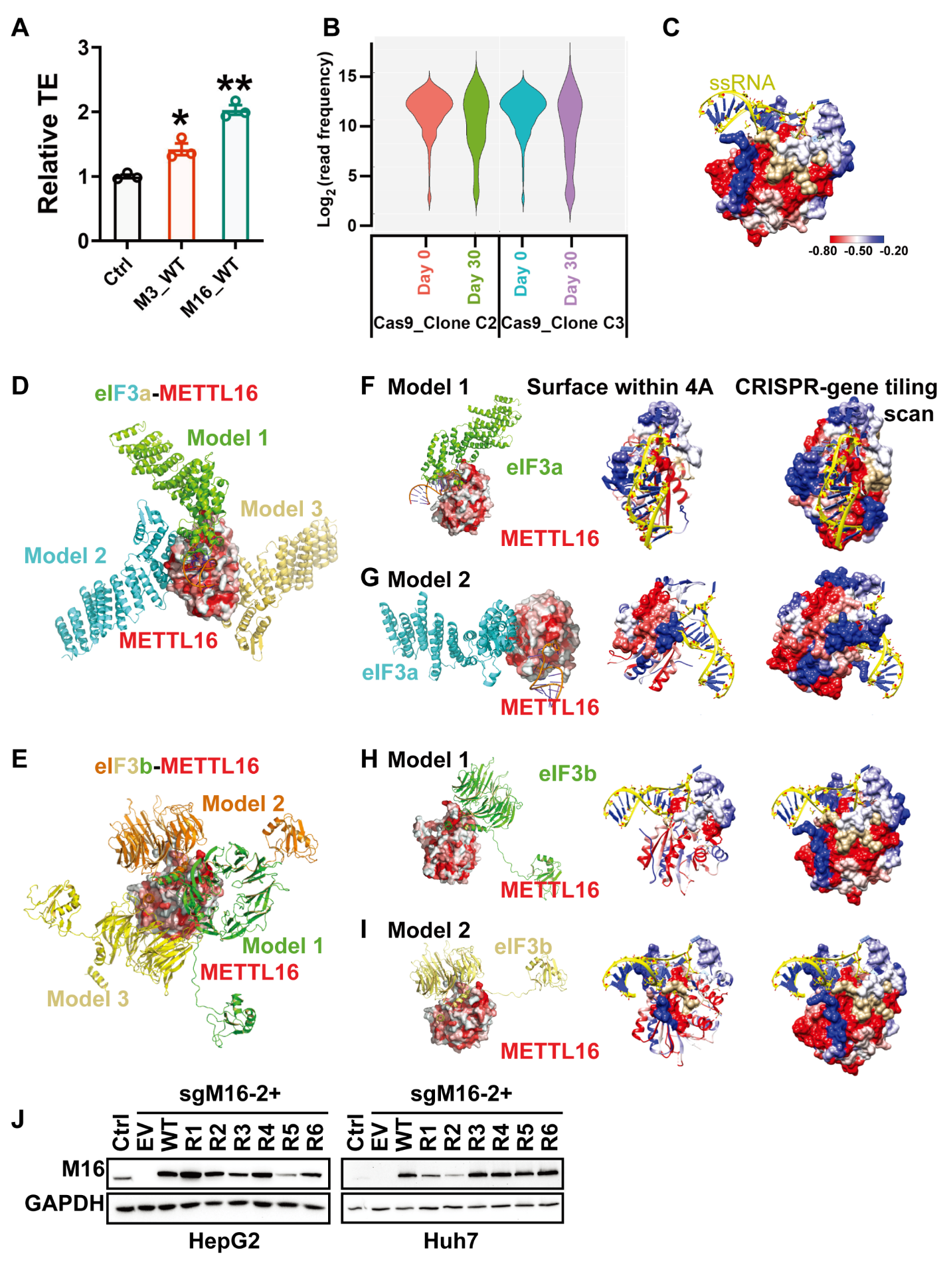
**

**Supplementary Figure 4. CRISPR tiling scan and protein-protein interaction (PPI) models pinpoint the essential elements of METTL16 responsible for its interaction with eIF3a/b.**

(A) RNA tethering assays showing the effect of ectopic expression of METTL3 (M3) or METTL16 (M16) on translation efficiency in HepG2 cells.

(B) The read frequency of CRISPR screening data from 2 groups of HepG2 Cas9 single clones on day 0 and day 30.

(C) Annotation of smoothed CRISPR screening relative to structural models of METTL16. ssRNA, single-stranded RNA.

(D) The best three PPI models for METTL16-eIF3a interaction.

(E) The best three PPI models for METTL16-eIF3b interaction.

(F) The PPI model 1 between eIF3a and METTL16 CRISPR gene tiling scan. Left, PPI modeled structure (model 1, M1); Middle, Visualization of METTL16 surface area within 4 amino acids (4A) from the predicted eIF3a-METTL16 model 1; Right, CRISPR scan plotting of METTL16 (methyltransferase domain, 1-310aa) from the predicted eIF3a-METTL16 model 1.

(G) The PPI model 2 between eIF3a and METTL16 CRISPR gene tiling scan.

(H) The PPI model 1 between eIF3b and METTL16 CRISPR gene tiling scan.

(I) The PPI model 2 between eIF3b and METTL16 CRISPR gene tiling scan.

(J) Representative Western blotting images showing the overexpression efficiency of wild-type or mutant METTL16 in Huh7 and HepG2 cells following *METTL16* KO.

Statistical analyses: un-paired *t-test* (A); **P* < 0.05, ***P* < 0.01.

# **Supplementary Figure 5**


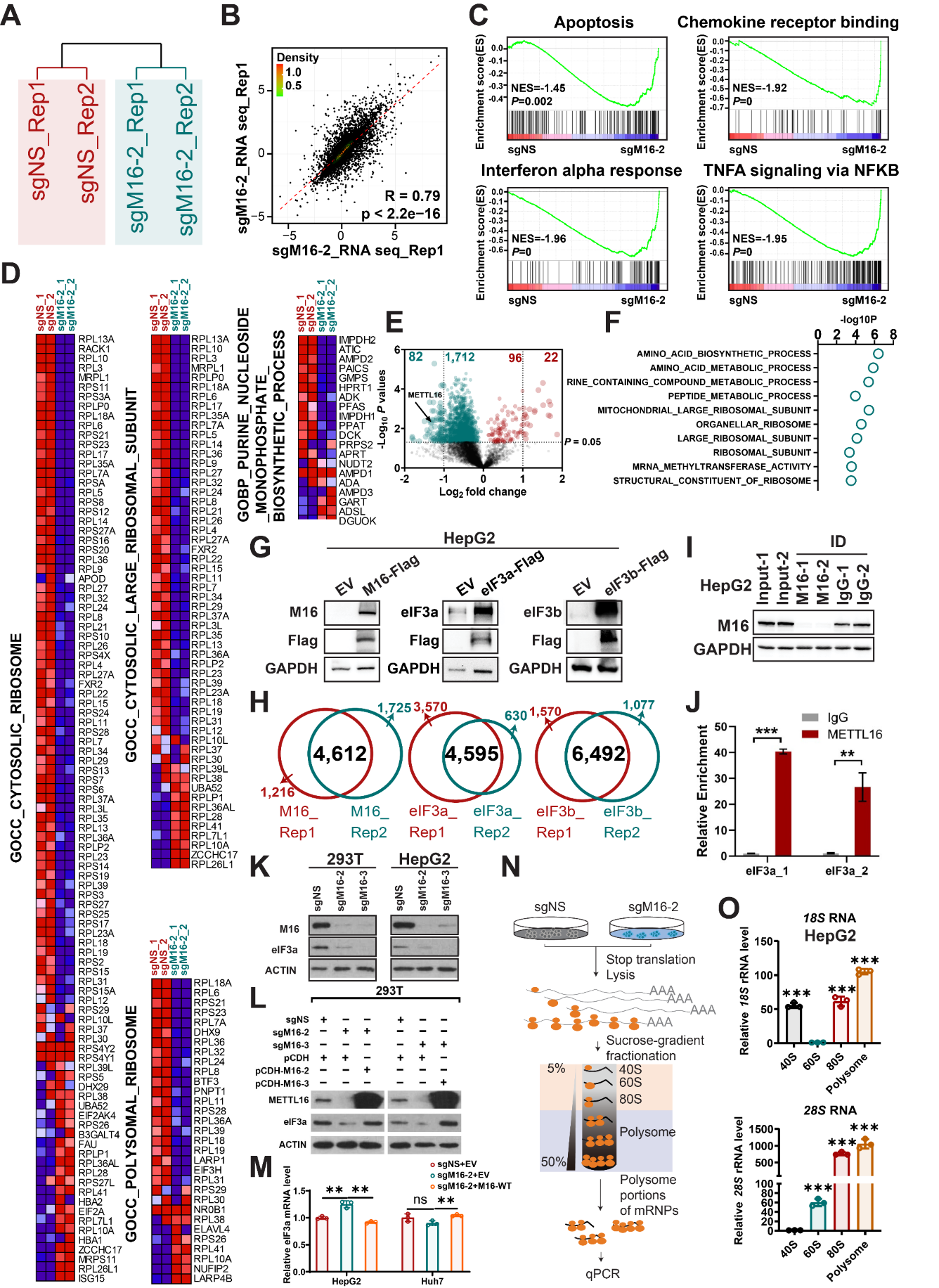


**Supplementary Figure 5. METTL16 enhances the translation-associated pathways and *eIF3a* mRNA translation efficiency in HCC.**

(A) Hierarchical clustering dendrogram of RNA-seq data from Huh7 cells upon *METTL16* KO.

(B) Scatterplot showing the high reproducibility of RNA-seq replicates of Huh7 cells upon *METTL16* KO. The Pearson correlation coefficients (R) of the normalized RNA-seq reads across the two replicates were calculated and displayed in the plots. A smoother regression line and 2D kernel density contour bands were also presented.

(C) GSEA enrichment of the top up-regulated genes upon *METTL16* depletion in Huh7.

(D) Heatmap showing the expression level of the core enriched genes in the indicated signal pathways. The results were derived from our RNA-seq data in Huh7 cells upon *METTL16* KO.

(E) MA plots displaying the decreased- and increased- expression proteins in HepG2 cells upon *METTL16* KO. The dashed vertical lines represent Log_2_(fold change) = 1 or -1. The significantly increased- or decreased- expression proteins are shown in red and blue, respectively (*P* < 0.05); the grey dots indicate *P* ≥ 0.05.

(F) GO enrichment analysis of the down-regulated proteins upon *METTL16* depletion in HepG2.

(G) Western blotting showing the overexpression efficiency of METTL16, eIF3a, and eIF3b in HepG2 cells for RIP-seq.

(H) Venn diagram showing the overlap of the specific bound transcripts between the two biological replicates of METTL16-, eIF3a-, or eIF3b-RIP-seq.

(I) Western blotting showing the METTL16 RIP efficiency in HepG2 cells. ID, Immunodepletion.

(J) METTL16 CLIP-qPCR analysis showing the interaction between METTL16 and *eIF3a* mRNA in HEK293T cells (n=3; mean ± SD).

(K) Western blotting showing the eIF3a protein level changes in HEK293T and HepG2 cells upon *METTL16* KO.

(L) Western blotting showing the eIF3a protein level changes in HEK293T cells upon *METTL16* KO and rescue expression.

(M) qPCR analysis of *eIF3a* mRNA level changes in HepG2 and Huh7 cells upon *METTL16* KO and rescue expression (n = 3; mean ± SD).

(N) Schematic describing the Ribo-qPCR assays in HepG2 cells with or without *METTL16* KO.

(O) Relative levels of 18S and 28S rRNAs in the 40S, 60S, 80S, and polysome fractions as determined by Ribo-qPCR.

Statistical analyses: Pearson’s correlation test (B), un-paired *t-test* (J, M, O); ns, not significant, ***P* < 0.01, ****P* < 0.001.

# **Supplementary Figure 6**

**
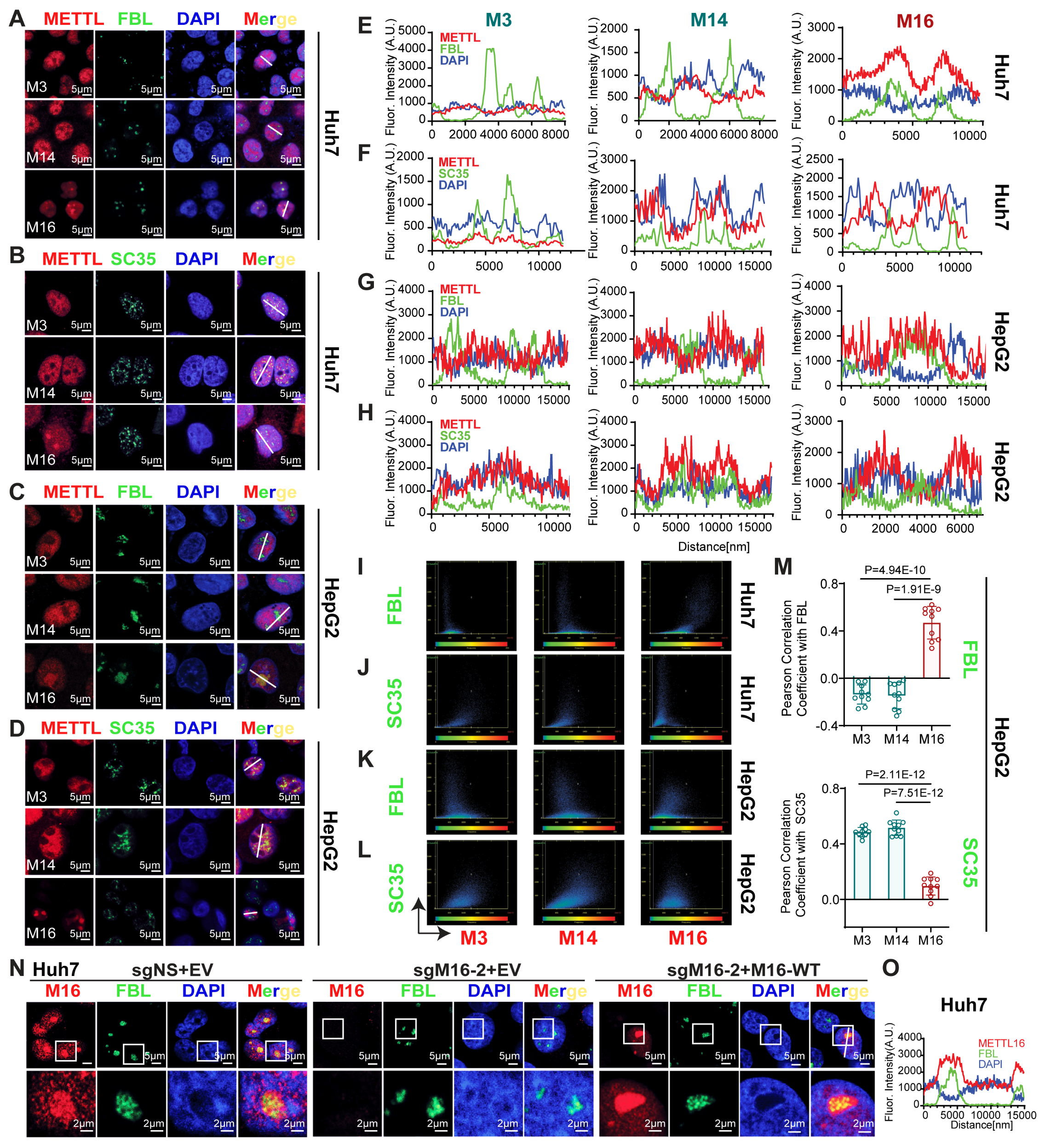
**

**Supplementary Figure 6. METTL16, but not METTL3 or METTL14, preferentially localizes to the nucleolus.**

(A) Representative confocal images of FBL (green) and 3 METTL members (red; including METTL3, M3; METTL14, M14; METTL16, M16) in the nucleus of Huh7 cells. FBL, nucleolar marker; DAPI, nuclear marker.

(B) Representative confocal images of SC35 (green) and METTL3/14/16(red; M3/14/16) in the nucleus of Huh7 cells. SC35, nuclear speckle marker.

(C) Representative confocal images of FBL and METTL3/14/16 in the nucleus of HepG2 cells.

(D) Representative confocal images of SC35 and METTL3/14/16 in the nucleus of HepG2 cells.

(E) Profile intensities of METTL3/14/16, FBL, and DAPI on the white arrows in Figure S6A.

(F) Profile intensities of METTL3/14/16, SC35, and DAPI on the white arrows in Figure S6B.

(G) Profile intensities of METTL3/14/16, FBL, and DAPI on the white arrows in Figure S6C.

(H) Profile intensities of METTL3/14/16, SC35, and DAPI on the white arrows in Figure S6D.

(I) Scatter plot analysis showing the colocalization between METTL3/14/16 and FBL in Huh7 cells.

(J) Scatter plot analysis showing the colocalization between METTL3/14/16 and SC35 in Huh7 cells.

(K) Scatter plot analysis showing the colocalization between METTL3/14/16 and FBL in HepG2 cells.

(L) Scatter plot analysis showing the colocalization between METTL3/14/16 and SC35 in HepG2 cells.

(M) Pearson’s correlation analysis between METTL3/14/16 and FBL (upper) or SC35 (lower) in HepG2 cells (n = 10; mean ± SD).

(N) Representative confocal images showing the subnuclear location of endogenous and rescue expression METTL16 in Huh7 cells. *METTL16* KO cells were used as negative control.

(O) Profile intensities of METTL16, FBL, and DAPI on the white arrows in Figure S6N.

Statistical analyses: un-paired *t-test* (M).

# **Supplementary Figure 7**

**
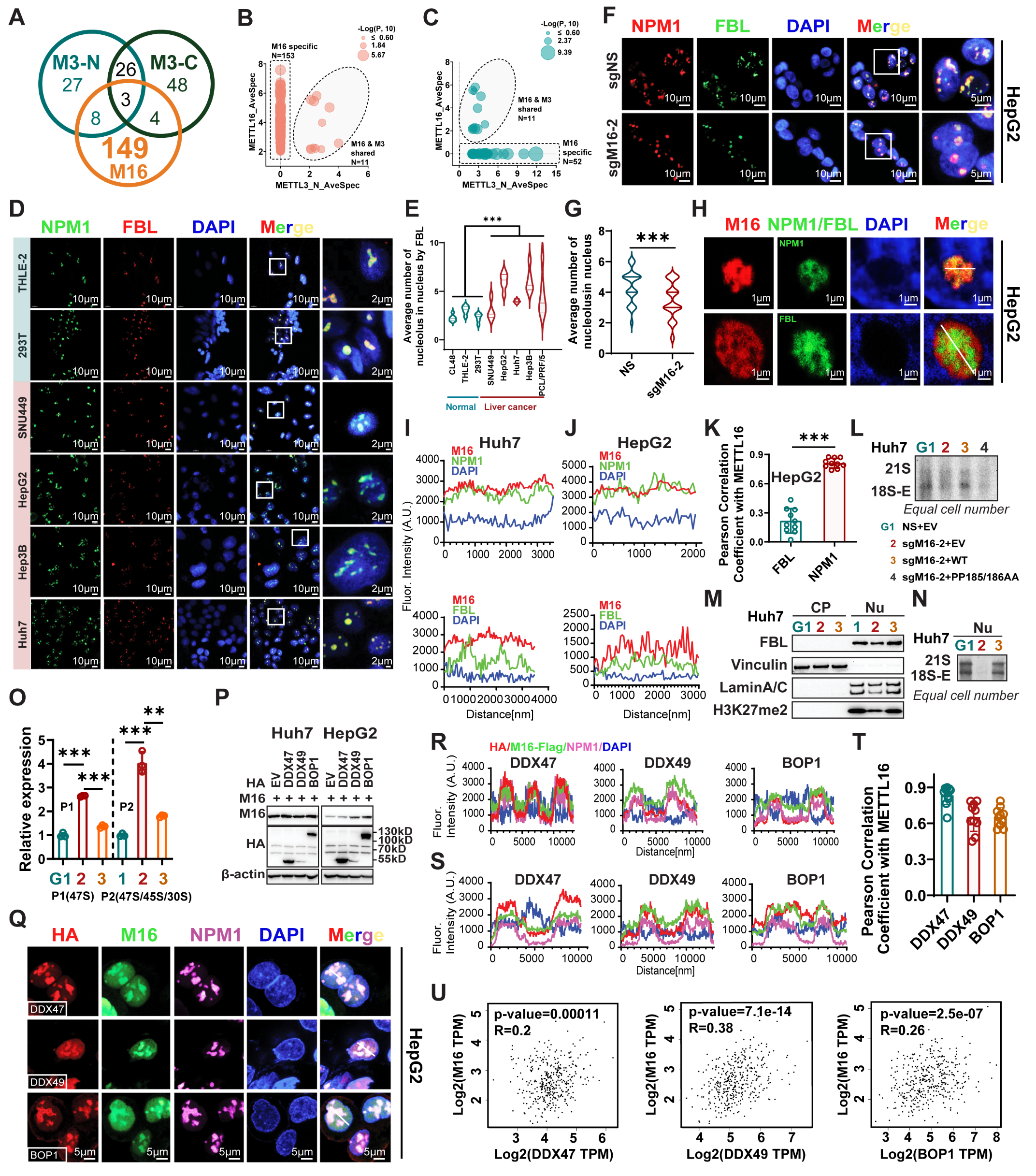
**

**Supplementary Figure 7. METTL16 preferentially localizes to the GC of the nucleolus and facilitates rRNA processing and ribosome biogenesis.**

(A) Venn diagram illustrating the overlap between METTL16- and METTL3-interacting proteins identified by BioID assay. M3-N, BirA*-METTL3 (BirA* tag at the N- terminus of METTL3); M3-C, METTL3-BirA* (BirA* tag at the C- terminus of METTL3); M16, METTL16-BirA* (BirA* tag at the C- terminus of METTL16).

(B-C) Bubble plot showing the METTL16-interacting (B) or METTL3-interacting (C) proteins identified by BioID assay. The size of each dot represents the *P* value of probability of binding of METTL16 or METTL3 with each protein. The proteins within the rectangle specifically interact with METTL16-BirA* (B) or BirA*-METTL3 (C); while the proteins within the oval interact with both METTL16 and BirA*-METTL3.

(D) Representative confocal images showing the nucleolus in normal (THLE-2 and HEK293T) and cancer (SNU449, HepG2, Hep3B and Huh7) cells.

(E) Statistical results of nucleolar numbers in normal cells and cancer cells (n＞40).

(F) Representative confocal images showing the effects of *METTL16* KO on nucleolar numbers in HepG2 cells.

(G) The statistical results showing the effects of *METTL16* KO on nucleolar numbers in HepG2 cells (n＞50).

(H) Representative confocal images showing the subnucleolar localization of METTL16 in HepG2 cells. FBL, DFC marker; NPM1, GC marker.

(I) Profile intensities of METTL16, NPM1, FBL, and DAPI on the white arrows in Huh7 cells in Figure 6K.

(J) Profile intensities of METTL16, NPM1, FBL, and DAPI on the white arrows in HepG2 cells in Figure S7H.

(K) Pearson’s correlation analysis showing the extent of colocalization of METTL16 with FBL or NPM1 in HepG2 cells (n = 10; mean ± SD).

(L) Representative images showing the effects of *METTL16* KO and rescued expression on 21S and 18S-E pre-rRNA levels as determined by Northern blotting in Huh7 cells.

(M) Representative Western blotting images showing the nuclear isolation efficiency in Huh7 cells. Vinculin was used as the loading control of cytoplasmic protein samples; FBL, Lamin A/C, and H3K27me2 were selected as the loading control of nuclear protein.

(N) Representative Northern blotting images showing the effects of *METTL16* KO and rescued expression on 30S, 21S, and 18S-E pre-rRNA levels in Huh7 nuclear.

(O) The effects of *METTL16* KO and rescued expression on pre-rRNA levels in Huh7 nuclear as determined by qPCR (n = 3; mean ± SD). P1 was used to detect 47S pre-rRNA; while P2 was used to detect 47S, 45S, and 30S pre-rRNAs.

(P) Representative Western blotting image showing the overexpression efficiency of METTL16, DDX47, DDX49, and BOP1 in Huh7 and HepG2 cells.

(Q) Representative confocal images showing the colocalization of METTL16 with DDX47, DDX49, or BOP1 in the nucleolus of HepG2 cells.

(R) Profile intensities of METTL16, DDX47 (or DDX49 or BOP1), NPM1, and DAPI on the white arrows in Huh7 cells in Figure 6Q.

(S) Profile intensities of METTL16, DDX47 (or DDX49 or BOP1), NPM1, and DAPI on the white arrows in HepG2 cells in Figure S7Q.

(T) Pearson’s correlation analysis showing the extent of colocalization of METTL16 with DDX47, DDX49, or BOP1 in HepG2 cells.

(U) The correlation between *METTL16* and *DDX47*, *DDX49* or *BOP1* in expression as detected by Pearson’s correlation analysis. All the raw data were downloaded from TCGA-LIHC database. *R* value and *P* value were displayed.

Statistical analyses: unpaired *t-test* (E, G, K, O), Pearson’s correlation test (U); ***P* < 0.01, ****P*<0.001.

# **Supplementary Table 1. CRISPR screening library sequences**

| **gRNA_id** | **sequence** |
| --- | --- |
| sg_hMETTL16_259_s | GAGACTCAACTATATTCACT |
| sg_hMETTL16_258_s | TGAGACTCAACTATATTCAC |
| hK_sg_hRPS20_90 | ACCAGTTCGAATGCCTACCA |
| sg_hMETTL16_149_a | TCAGAGCTCTGACTGCTTCG |
| sg_hMETTL16_178_s | TCTGACGTGTACTCTCCTAA |
| sg_hMETTL16_275_s | CACTGGGTAGAAGATCTGAT |
| sg_hMETTL16_1591_a | AACCCAGTGCATCTCCACTA |
| hK_sg_hMYC_1138 | ACAACGTCTTGGAGCGCCAG |
| hK_sg_RPL23A_2 | GTCGCAGTGTCTTCGGCCGC |
| hK_sg_hBRD4_309 | TAAGATCATTAAAACGCCTA |
| sg_hMETTL16_371_s | TTACTTGGAGCAACCTTGAA |
| sg_hMETTL16_323_s | CTCCGAAGAGGAATTGACAT |
| hK_sg_PCNA_2 | CTACCGCTGCGACCGCAACC |
| sg_hMETTL16_234_a | TTGAGTCTCAAGGGAACTGT |
| hK_sg_PRL9_1 | GGACGCACAGTTATCGTGAA |
| sg_hMETTL16_774_a | ATGCGAAGCTCCTCCTTCAG |
| sg_hMETTL16_1600_s | TGCCTTAGTGGAGATGCACT |
| sg_hMETTL16_1413_s | AAAGGAGTGAGGAAAAGGGA |
| sg_hMETTL16_729_a | AGCATGCAGCTATACCATCT |
| sg_hMETTL16_844_s | TCAAGGTCGGACAATGAGAT |
| sg_hMETTL16_235_a | GTTGAGTCTCAAGGGAACTG |
| hK_sg_hRPS20_172 | AAGCCGCAACGTAAAATCCT |
| sg_hMETTL16_1412_s | GAAAGGAGTGAGGAAAAGGG |
| hK_sg_PRL9_2 | AATGTAGAACTCAGCCTTCT |
| hK_sg_POLR2A_1 | AAGCGAATGTCTGTGACGGA |
| sg_hMETTL16_356_a | TCAAGGTTGCTCCAAGTAAG |
| sg_hMETTL16_211_s | ACTTTCTATTGATATTCCAT |
| sg_hMETTL16_177_s | CTCTGACGTGTACTCTCCTA |
| sg_hMETTL16_216_a | GTGGGAATTAGTCTCTCCAA |
| sg_hMETTL16_373_a | GAGGAAATACCAGCCATTCA |
| sg_hMETTL16_621_a | CCTGTATTAACAGAACTAGG |
| sg_hMETTL16_1111_s | GGAAGTCAGCCTTTTCCTAA |
| sg_hMETTL16_500_a | CTTCTTTAAGAGCATCCATC |
| hK_sg_PCNA_1 | GGACTCGTCCCACGTCTCTT |
| hK_sg_RPA3_1 | CCGGCGTTGATGCGCGACCT |
| sg_hMETTL16_470_a | TCTGTGGCACTTTCACCACT |
| sg_hMETTL16_151_a | CGTCAGAGCTCTGACTGCTT |
| sg_hMETTL16_792_s | AGGAGGAGCTTCGCATACAA |
| sg_hMETTL16_843_s | GTCAAGGTCGGACAATGAGA |
| hK_sg_RPA3_2 | GCCGGCGTTGATGCGCGACC |
| sg_hMETTL16_1128_s | TAACGGCCATAGAAAACTCC |
| sg_hMETTL16_743_s | AGATGGTATAGCTGCATGCT |
| sg_hMETTL16_1607_s | GTGGAGATGCACTGGGTTGA |
| sg_hMETTL16_496_s | GCCACAGAAGACACTCCTGA |
| sg_hMETTL16_356_s | TCTTGCATCTACCCCTTACT |
| sg_hMETTL16_557_a | ATTGATTGGCAAAAAAGGGA |
| sg_hMETTL16_1417_s | GAGTGAGGAAAAGGGAGGGG |
| hK_sg_POLR2D_2 | TGGGCAAAGGTTGGCCAAAC |
| sg_hMETTL16_1618_s | CTGGGTTGAGGGCCAGAACA |
| sg_hMETTL16_182_a | AAAGTCCAAAATCTTCCCTT |
| sg_hMETTL16_1640_a | TACGTATGTAGGTGCAAAGC |
| sg_hMETTL16_793_s | GGAGGAGCTTCGCATACAAG |
| sg_hMETTL16_831_s | ACACTGAATTCTGTCAAGGT |
| sg_hMETTL16_827_s | ACGTACACTGAATTCTGTCA |
| sg_hMETTL16_791_s | AAGGAGGAGCTTCGCATACA |
| hK_sg_hBRD4_336 | GGGAACAATAAAGAAGCGCT |
| sg_hMETTL16_1619_a | GGTTCATCAGATCCCTGTTC |
| sg_hMETTL16_1599_s | ATGCCTTAGTGGAGATGCAC |
| sg_hMETTL16_808_a | GAATTCAGTGTACGTTACTT |
| sg_hMETTL16_397_s | GTATTTCCTCGCAACAGAAG |
| sg_hMETTL16_772_s | ATGCAGCCTGGCGCCTCTGA |
| sg_hMETTL16_635_s | CCTAGTTCTGTTAATACAGG |
| sg_hMETTL16_358_a | ATTCAAGGTTGCTCCAAGTA |
| hK_sg_hMYC_122 | GCCGTATTTCTACTGCGACG |
| sg_hMETTL16_1082_s | CAGCATAAACGAGTTCCCTG |
| sg_hMETTL16_662_s | ACAGAGATCATGGCAGAAGG |
| hK_sg_CDK1_2 | AATCCATGTACTGACCAGGA |
| sg_hMETTL16_311_s | GACAAAAGTACTCTCCGAAG |
| sg_hMETTL16_632_s | CCGCCTAGTTCTGTTAATAC |
| sg_hMETTL16_1570_s | TTTGATAAACGTTAAGAAGG |
| sg_hMETTL16_798_s | AGCTTCGCATACAAGGGGTG |
| sg_hMETTL16_659_s | ATCACAGAGATCATGGCAGA |
| sg_hMETTL16_1123_a | ATGAATCCAGGAGTTTTCTA |
| sg_hMETTL16_624_a | CCTCCTGTATTAACAGAACT |
| sg_hMETTL16_1506_s | GCAGCCCAGTGGCTGAAAGG |
| sg_hMETTL16_1414_s | AAGGAGTGAGGAAAAGGGAG |
| sg_hMETTL16_1294_a | CCGCAGAGCAGGCCCACAGG |
| sg_hMETTL16_652_s | AGGAGGCATCACAGAGATCA |
| sg_hMETTL16_128_a | GGTCTTTAAAATTAAGGCTG |
| sg_hMETTL16_1495_s | TGAGCAGTTCGGCAGCCCAG |
| sg_hMETTL16_1363_s | GAGCCAGGAGTCCCTGTCCC |
| sg_hMETTL16_64_s | ACCTCCTGACTTTGCATATC |
| sg_hMETTL16_57_a | GATGCCAGATATGCAAAGTC |
| sg_hMETTL16_37_s | GCATGCAAGAAATAGATACA |
| sg_hMETTL16_1617_s | ACTGGGTTGAGGGCCAGAAC |
| hK_sg_POLR2A_2 | CAGGGGGTGATTGAGCGGAC |
| sg_hMETTL16_1409_s | GATGAAAGGAGTGAGGAAAA |
| sg_hMETTL16_357_a | TTCAAGGTTGCTCCAAGTAA |
| sg_hMETTL16_1276_s | AGAACTGGCCAGGGGCCCCC |
| sg_hMETTL16_742_s | TAGATGGTATAGCTGCATGC |
| sg_hMETTL16_1606_s | AGTGGAGATGCACTGGGTTG |
| sg_hMETTL16_760_s | GCTGGGAAAGAAATGCAGCC |
| hK_sg_CDK9_1 | CTGGCCGGTCTTGCGGTGCC |
| sg_hMETTL16_775_s | CAGCCTGGCGCCTCTGAAGG |
| sg_hMETTL16_1408_s | GGATGAAAGGAGTGAGGAAA |
| sg_hMETTL16_807_a | AATTCAGTGTACGTTACTTT |
| sg_hMETTL16_127_s | TCTGAATGGAAGAGTGAGGT |
| sg_hMETTL16_1109_a | TTTCTATGGCCGTTAGGAAA |
| sg_hMETTL16_1281_a | CCACAGGGGGTCCTCTCCTG |
| sg_hMETTL16_856_a | ATCATAAAAACTCCAAGCTA |
| sg_hMETTL16_1282_a | CCCACAGGGGGTCCTCTCCT |
| sg_hMETTL16_1240_a | GCTATTGCCAGACTCTTTGG |
| sg_hMETTL16_1195_s | AGAAGTTCCCCGAGCTCCTG |
| sg_hMETTL16_961_a | GAGGGATAATTCCTTCATCA |
| sg_hMETTL16_1310_s | TGTGGGCCTGCTCTGCGGGA |
| sg_hMETTL16_1296_a | TCCCGCAGAGCAGGCCCACA |
| sg_hMETTL16_113_s | CATGTTCAGATAAATCTGAA |
| sg_hMETTL16_1295_a | CCCGCAGAGCAGGCCCACAG |
| sg_hMETTL16_1504_s | CGGCAGCCCAGTGGCTGAAA |
| sg_hMETTL16_1366_s | CCAGGAGTCCCTGTCCCAGG |
| sg_hMETTL16_1070_a | CACAGGGAACTCGTTTATGC |
| sg_hMETTL16_1280_a | CACAGGGGGTCCTCTCCTGG |
| sg_hMETTL16_1537_a | ACACTTAAACAGGTACTGTC |
| sg_hMETTL16_1573_s | GATAAACGTTAAGAAGGAGG |
| sg_hMETTL16_1451_s | AGTTGTCAAGGCTCTAGCAA |
| sg_hMETTL16_1484_s | CAAGAGGCTTCTGAGCAGTT |
| sg_hMETTL16_314_a | TACCTATGTCAATTCCTCTT |
| sg_hMETTL16_1330_a | CGGGCACGGGCCCTCCACAG |
| sg_hMETTL16_244_a | GTGAATATAGTTGAGTCTCA |
| sg_hMETTL16_1439_s | GAGGTTTTGGAAAGTTGTCA |
| sg_hMETTL16_1292_s | CCCCAGGAGAGGACCCCCTG |
| sg_hMETTL16_1297_a | TTCCCGCAGAGCAGGCCCAC |
| hK_sg_POLR2D_1 | TGAGAGTGCAGAGGACGAAC |
| sg_hMETTL16_1293_s | CCCAGGAGAGGACCCCCTGT |
| sg_hMETTL16_1526_a | GGTACTGTCCGGCCACTCCT |
| sg_hMETTL16_1242_a | TGGCTATTGCCAGACTCTTT |
| sg_hMETTL16_1281_s | TGGCCAGGGGCCCCCAGGAG |
| sg_hMETTL16_1503_s | TCGGCAGCCCAGTGGCTGAA |
| sg_hMETTL16_1389_a | TCACTCCTTTCATCCTCCGT |
| sg_hMETTL16_1330_s | AGGCGAGGCTGCCGCTGTGG |
| sg_hMETTL16_1283_a | GCCCACAGGGGGTCCTCTCC |
| sg_hMETTL16_1306_s | CCCCTGTGGGCCTGCTCTGC |
| sg_hMETTL16_1402_s | CACGGAGGATGAAAGGAGTG |
| sg_hMETTL16_1384_s | GGAGGAAAACCCGGAACCCA |
| sg_hMETTL16_1029_s | GCATAGTCGTTGTCACGACA |
| sg_hMETTL16_1395_s | CGGAACCCACGGAGGATGAA |
| sg_hMETTL16_1331_s | GGCGAGGCTGCCGCTGTGGA |
| sg_hMETTL16_1200_a | AAGGCCTGAATGACGTCCTC |
| sg_hMETTL16_979_a | GCGCAGAGGTGATGCTTTGA |
| sg_hMETTL16_1468_s | CAACGGAGCCCAGGACCAAG |
| sg_hMETTL16_1207_s | AGCTCCTGAGGACGTCATTC |
| sg_hMETTL16_855_s | CAATGAGATGGGCCTTAGCT |
| sg_hMETTL16_1268_s | AATAGCCAAGAACTGGCCAG |
| sg_hMETTL16_1420_s | TGAGGAAAAGGGAGGGGTGG |
| sg_hMETTL16_486_a | TCCATCAGGAGTGTCTTCTG |
| sg_hMETTL16_1315_s | GCCTGCTCTGCGGGAAGGCG |
| sg_hMETTL16_123_s | TAAATCTGAATGGAAGAGTG |
| sg_hMETTL16_940_s | GAGAAAACCCATAACATTCG |
| sg_hMETTL16_1343_a | GGGACTCCTGGCTCGGGCAC |
| sg_hMETTL16_558_a | AATTGATTGGCAAAAAAGGG |
| sg_hMETTL16_1191_a | ATGACGTCCTCAGGAGCTCG |
| sg_hMETTL16_1193_a | GAATGACGTCCTCAGGAGCT |
| sg_hMETTL16_1192_a | AATGACGTCCTCAGGAGCTC |
| sg_hMETTL16_433_s | CAACTATGCAAAGAAAAATG |
| sg_hMETTL16_290_a | GAGTACTTTTGTCAGAATCC |
| sg_hMETTL16_1349_a | GGGACAGGGACTCCTGGCTC |
| sg_hMETTL16_1244_s | AAGCCCACCCCCAAAGAGTC |
| sg_hMETTL16_464_s | AACTTATCTGATCTCATAAA |
| sg_hMETTL16_994_s | CAAAGCATCACCTCTGCGCT |
| sg_hMETTL16_375_s | TTGGAGCAACCTTGAATGGC |
| hK_sg_CDK9_2 | GATCTCCCGCAAGGCTGTAA |
| sg_hMETTL16_1390_a | CTCACTCCTTTCATCCTCCG |
| sg_hMETTL16_1327_s | GGAAGGCGAGGCTGCCGCTG |
| sg_hMETTL16_1387_s | GGAAAACCCGGAACCCACGG |
| sg_hMETTL16_1499_a | GTTTCCCCCTTTCAGCCACT |
| hK_sg_CDK1_1 | GGGTTCCTAGTACTGCAATT |
| sg_hMETTL16_54_a | GCCAGATATGCAAAGTCAGG |
| sg_hMETTL16_1527_a | AGGTACTGTCCGGCCACTCC |
| sg_hMETTL16_618_a | GTATTAACAGAACTAGGCGG |
| sg_hMETTL16_1344_a | AGGGACTCCTGGCTCGGGCA |
| sg_hMETTL16_961_s | GGTGCTGGCGTCCGTGATGA |
| sg_hMETTL16_1500_a | CGTTTCCCCCTTTCAGCCAC |
| sg_hMETTL16_1350_a | TGGGACAGGGACTCCTGGCT |
| sg_hMETTL16_1241_a | GGCTATTGCCAGACTCTTTG |
| sg_hMETTL16_1262_a | GGGGGCCCCTGGCCAGTTCT |
| sg_hMETTL16_1465_a | CTCAGAAGCCTCTTGGTCCT |
| sg_hMETTL16_79_a | CTGCTTAAAATCTGGATATT |
| sg_hMETTL16_1375_s | CCTGTCCCAGGAGGAAAACC |
| sg_hMETTL16_1267_s | CAATAGCCAAGAACTGGCCA |
| sg_hMETTL16_1520_s | GAAAGGGGGAAACGTCTCCC |
| sg_hMETTL16_1369_a | GGGTTCCGGGTTTTCCTCCT |
| sg_hMETTL16_1466_a | GCTCAGAAGCCTCTTGGTCC |
| sg_hMETTL16_684_s | GTGAATTAGAGTTTGTTAAA |
| sg_hMETTL16_1588_s | GGAGGTGGACGATGCCTTAG |
| sg_hMETTL16_1363_a | CGGGTTTTCCTCCTGGGACA |
| sg_hMETTL16_1087_a | GCTGACTTCCTCTTTTCCAC |
| sg_hMETTL16_1086_a | CTGACTTCCTCTTTTCCACA |
| sg_hMETTL16_1219_a | GGTGGGCTTTTTCTCTTCCA |
| sg_hMETTL16_1651_a | GAAAATTTGGTTACGTATGT |
| sg_hMETTL16_1243_a | TTGGCTATTGCCAGACTCTT |
| sg_hMETTL16_609_a | GAACTAGGCGGAGGTCTTCG |
| sg_hMETTL16_1383_a | CTTTCATCCTCCGTGGGTTC |
| sg_hMETTL16_767_a | GCTCCTCCTTCAGAGGCGCC |
| sg_hMETTL16_1505_s | GGCAGCCCAGTGGCTGAAAG |
| sg_hMETTL16_936_a | GCCAGCACCACGAATGTTAT |
| sg_hMETTL16_1305_s | CCCCCTGTGGGCCTGCTCTG |
| sg_hMETTL16_1003_s | ACCTCTGCGCTCGGAGACGG |
| sg_hMETTL16_1355_a | CCTCCTGGGACAGGGACTCC |
| sg_hMETTL16_1135_a | CTTTCTCCTTAAATGAATCC |
| sg_hMETTL16_1090_s | ACGAGTTCCCTGTGGAAAAG |
| sg_hMETTL16_134_a | CTTCGGGGTCTTTAAAATTA |
| sg_hMETTL16_283_s | AGAAGATCTGATCGGTCACC |
| sg_hMETTL16_243_a | TGAATATAGTTGAGTCTCAA |
| sg_hMETTL16_701_a | TTTTAAGTTGTAGACTGTCA |
| sg_hMETTL16_1140_s | AAAACTCCTGGATTCATTTA |
| sg_hMETTL16_980_a | AGCGCAGAGGTGATGCTTTG |
| sg_hMETTL16_571_a | TACCTTGGCTTCCAATTGAT |
| sg_hMETTL16_150_a | GTCAGAGCTCTGACTGCTTC |
| sg_hMETTL16_1000_s | ATCACCTCTGCGCTCGGAGA |
| sg_hMETTL16_1382_a | TTTCATCCTCCGTGGGTTCC |
| sg_hMETTL16_1236_a | TTGCCAGACTCTTTGGGGGT |
| sg_hMETTL16_1237_a | ATTGCCAGACTCTTTGGGGG |
| sg_hMETTL16_1567_s | GTGTTTGATAAACGTTAAGA |
| sg_hMETTL16_1370_a | TGGGTTCCGGGTTTTCCTCC |
| sg_hMETTL16_188_s | ACTCTCCTAAGGGAAGATTT |
| sg_hMETTL16_1364_a | CCGGGTTTTCCTCCTGGGAC |
| sg_hMETTL16_1057_s | AAAAATTCTCACTGATTTGA |
| sg_hMETTL16_1472_a | CGAACTGCTCAGAAGCCTCT |
| sg_hMETTL16_927_a | ACGAATGTTATGGGTTTTCT |
| sg_hMETTL16_392_a | TATCATCCACTTCTGTTGCG |
| sg_hMETTL16_897_a | AATTTTCTTCGCTTACTTGG |
| CN_sg_Luc_474 | GATTCTAAAACGGATTACCA |
| CN_sg_Ren_170 | ATGTCGCCATAAATAAGAAG |
| sg_hMETTL16_1426_s | AAAGGGAGGGGTGGAGGTTT |
| sg_hMETTL16_1305_a | GCCTCGCCTTCCCGCAGAGC |
| sg_hMETTL16_1664_a | CTGCAACAAGCCTGAAAATT |
| sg_hMETTL16_87_a | TGAACATGCTGCTTAAAATC |
| hK_sg_RPL23A_1 | TGCGGATCTTCTTCTTTTTG |
| sg_hMETTL16_1273_a | GGTCCTCTCCTGGGGGCCCC |
| sg_hMETTL16_946_s | ACCCATAACATTCGTGGTGC |
| CN_sg_Neg_3 | GGCAGTCGTTCGGTTGATAT |
| CN_sg_LacZ_904 | AATCCCGAATCTCTATCGTG |
| CN_sg_Ren_55 | GGATGATAACTGGTCCGCAG |
| CN_sg_LacZ_336 | GTGAGCGAGTAACAACCCGT |
| CN_sg_Neg_1 | GTAGCGAACGTGTCCGGCGT |
| sg_hMETTL16_1266_s | GCAATAGCCAAGAACTGGCC |
| sg_hMETTL16_571_s | TCCCTTTTTTGCCAATCAAT |
| sg_hMETTL16_1213_s | TGAGGACGTCATTCAGGCCT |
| sg_hMETTL16_1115_a | AGGAGTTTTCTATGGCCGTT |
| CN_sg_Ren_681 | GAAATCCCGTTAGTAAAAGG |
| sg_hMETTL16_937_a | CGCCAGCACCACGAATGTTA |
| sg_hMETTL16_900_a | TCTAATTTTCTTCGCTTACT |
| CN_sg_Neg_7 | GATACACGAAGCATCACTAG |
| sg_hMETTL16_1348_s | GGAGGGCCCGTGCCCGAGCC |
| CN_sg_LacZ_1739 | AACCCGTGGTCGGCTTACGG |
| CN_sg_Neg_2 | GACCGGAACGATCTCGCGTA |
| CN_sg_Ren_794 | GGCACCTTCAACAATAGCAT |
| sg_hMETTL16_562_a | TTCCAATTGATTGGCAAAAA |
| sg_hMETTL16_1665_s | ACATACGTAACCAAATTTTC |
| CN_sg_Luc_560 | TGTGCCAGAGTCCTTCGATA |
| CN_sg_Luc_1451 | GTGCTCCAAAACAACAACGG |
| CN_sg_Luc_1058 | AGCTATTCTGATTACACCCG |
| CN_sg_LacZ_1725 | ATGATGAAAACGGCAACCCG |
| CN_sg_Luc_1158 | ACGCTGGGCGTTAATCAAAG |
| CN_sg_Luc_371 | GGGCATTTCGCAGCCTACCG |
| CN_sg_Ren_649 | TCACGAGGCCATGATAATGT |
| CN_sg_Ren_58 | TTACATCTGGCCCACCACTG |
| sg_hMETTL16_1459_s | AGGCTCTAGCAACGGAGCCC |
| CN_sg_LacZ_1659 | ACGAAGCCGCCCTGTAAACG |
| sg_hMETTL16_580_s | TGCCAATCAATTGGAAGCCA |
| CN_sg_Neg_10 | GTCGTGAAGTGCATTCGATC |
| CN_sg_Neg_6 | GTCATACATGGATAAGGCTA |
| CN_sg_LacZ_1583 | TCGCGTGGGCGTATTCGCAA |
| CN_sg_Luc_1539 | ACCGCGAAAAAGTTGCGCGG |
| CN_sg_LacZ_2496 | TGATTACGACCGCTCACGCG |
| CN_sg_Ren_307 | ATTACAAATATCTTACTGCA |
| CN_sg_Ren_664 | TTTACTAACGGGATTTCACG |
| CN_sg_Neg_4 | GCTTGAGCACATACGCGAAT |
| CN_sg_LacZ_838 | CAGACGTAGTGTGACGCGAT |
| CN_sg_Neg_9 | GATCCATGTAATGCGTTCGA |
| CN_sg_LacZ_907 | CCCGAATCTCTATCGTGCGG |
| CN_sg_Luc_1536 | ACAACCGCGAAAAAGTTGCG |
| CN_sg_Luc_650 | GGCATGCGAGAATCTCACGC |
| CN_sg_Ren_211 | GGTATAATACACCGCGCTAC |
| sg_hMETTL16_1261_s | GTCTGGCAATAGCCAAGAAC |
| CN_sg_LacZ_1465 | CATCGGGCAAATAATATCGG |
| CN_sg_Luc_1189 | ACATAACCGGACATAATCAT |
| sg_hMETTL16_561_a | TCCAATTGATTGGCAAAAAA |
| CN_sg_Neg_8 | GAACGTTGGCACTACTTCAC |
| CN_sg_Ren_158 | TATTTTTTTACATGGTAACG |
| sg_hMETTL16_331_s | TTTTATTTTATTAGGCACGG |
| sg_hMETTL16_1547_a | CGTTTATCAAACACTTAAAC |
| sg_hMETTL16_330_s | TTTTTATTTTATTAGGCACG |
| sg_hMETTL16_329_s | ATTTTTATTTTATTAGGCAC |
| CN_sg_Neg_5 | GTGGTAGAATAACGTATTAC |
| sg_hMETTL16_993_a | TCCGCCGTCTCCGAGCGCAG |
| sg_hMETTL16_328_s | TATTTTTATTTTATTAGGCA |
| sg_hMETTL16_723_s | TACAACTTAAAAAAAGATTA |
| sg_hMETTL16_1007_s | CTGCGCTCGGAGACGGCGGA |
| sg_hMETTL16_1525_s | GGGGAAACGTCTCCCAGGAG |
| sg_hMETTL16_1529_s | AAACGTCTCCCAGGAGTGGC |

#

# **Supplementary Table 2. List of oligonucleotides**

| **Primer name** | **Sequence** | **Research purpose** |  |
| --- | --- | --- | --- |
| P1 47S-F | GCTGACACGCTGTCCTCTGG | RT-qPCR |  |
| P1 47S-R | GCTAGCCGGGTCACCGGTAG | RT-qPCR |  |
| P2 5' ETS-F | CCTTCCCCAGGCGTCCCTCG | RT-qPCR |  |
| P2 5' ETS-R | GGCAGCGCTACCATAACGGA | RT-qPCR |  |
| Human ACTIN-F | CACTCTTCCAGCCTTCCTTC | RT-qPCR |  |
| Human ACTIN-R | GTACAGGTCTTTGCGGATGT | RT-qPCR |  |
| Human GAPDH-F | TCAACGACCACTTTGTCAAGCTCA | RT-qPCR |  |
| Human GAPDH-R | GCTGGTGGTCCAGGGGTCTTACT | RT-qPCR |  |
| Human eIF3a-F | ACTCAGGATCGTACTGACAGATT | RT-qPCR |  |
| Human eIF3a-R | ATGGTACAGGCGCTCTACTCT | RT-qPCR |  |
| Human 18S-F | CTGCCCTATCAACTTTCGATGGTAG | RT-qPCR | |
| Human 18S-R | CCGTTTCTCAGGCTCCCTCTC | RT-qPCR | |
| Human 28S-F | TGTCGGCTCTTCCTATCATTGT | RT-qPCR | |
| Human 28S-R | ACCCAGCTCACGTTCCCTATTA | RT-qPCR | |
| Human eIF3a-RIP-1-F | ATTCGTAATGCGACTCAAAGCTGC | CLIP-qPCR |  |
| Human eIF3a-RIP-1-R | AGTATGTTATCCTGCGTTCTTC | CLIP-qPCR |  |
| Human eIF3a-RIP-2-F | ATGATGAAGATAGAGAGCCCTC | CLIP-qPCR |  |
| Human eIF3a-RIP-2-R | AGAGAACCTATCTTCCTCAGG | CLIP-qPCR |  |
| Human eIF3a_F_pSIN4_In fusion | TTCTTCCATTTCAGGTGTCGTGAGGAATTC | Construction of pSIN4-eIF3a |  |
| Human eIF3b_R_pSIN4_In fusion | CATGCGGATCCTTCGAACTAGTTTACGCGT |  |  |
| Human eIF3b-F-pSIN4_In fusion | CTAGTTCGAAGGATCCGGCCGCCCAGGACGCGGAGAACGTGGC | Construction of pSIN4-eIF3b |  |
| Human eIF3b-R-PSIN4_In fusion | AGATGCATGCGGATCCTCACTCCTGATTCCCGAGGGGAA |  |  |
| Human METTL16-msg2-E90A-G94A_F | GCAGCTGCGGCCGCTCACCAGGATTCTGACAAAAG | Construction of mutant METTL16 |  |
| Human METTL16-msg2-E90A-G94A_R | TACCCAGTGGATGTAATTCAAGCGGCCGCAGCTGC |  |  |
| Human METTL16-GW126/127AA_F | AATGCCGCGTACTTTTTGGCTACCGAAGTG |  |  |
| Human METTL16-GW126/127AA_R | AAAGTACGCGGCATTCAAGGTTGCTCCAAG |  |  |
| Human METTL16-EII175-177AAA_F | TCT GCG GCA GCC TAT GAC TTT TGC ATG TGC |  |  |
| Human METTL16-EII175-177AAA_R | ATA GGC TGC CGC AGA TTC TTC TTT AAG AGC |  |  |
| Human METTL16-VNT210-212AAA _F | GCTGCTGCAGCAGGCATCACAGAGATCATGGC |  |  |
| Human METTL16-VNT210-212AAA _R | TGCTGCAGCAGCAGAACTAGGCGGAGGTCTTC |  |  |
| Human METTL16-R243A _F | AGA TTA GCA TGG TAT AGC TGC ATG CTG GGA |  |  |
| Human METTL16-R243A _R | ATA CCA TGC TAA TCT TTT TTT AAG TTG TAG ACT GTC ATG |  |  |
| Human METTL16-Y290A _F | AGT TTT GCT GAT GAT GTC ACA GTA CCA TCA CCA |  |  |
| Human METTL16-Y290A _R | ATC ATC AGC AAA ACT CCA AGC TAA GGC CCA |  |  |
| Human DDX47_F | GCGGCACCCGAGGAACACGAT | Construction of DDX47 |  |
| Human DDX47_R | TTAACGGCCTTTCCGCTTCTT |  |  |
| Human pmiRNA1-XbaI-HA-DDX47-F | CATAGAAGATTCTAGAGCC ACC ATG TACCCATACGACGTCCCAGACTACGCT GCGGCACCCGAGGAACACGAT |  |  |
| Human pmiRNA1-Not1-DDX47-R | AGATCCTTCGCGGCCGC TTAACGGCCTTTCCGCTTCTT |  |  |
| Human DDX49_F | GCAGGCTTCGCGGAGCTCGGG | Construction of DDX49 |  |
| Human DDX49_R | TCAGACCAGGCCCTGGGAGGGG |  |  |
| Human pmiRNA1-XbaI-HA-DDX49-F | CATAGAAGATTCTAGAGCC ACC ATG TACCCATACGACGTCCCAGACTACGCT GCAGGCTTCGCGGAGCTCGGG |  |  |
| Human pmiRNA1-Not1-DDX49-R | AGATCCTTCGCGGCCGC TCAGACCAGGCCCTGGGAGGGG |  |  |
| Human BOP1_F | GCGGGTTCGCGGGGTGCGG | Construction of BOP1 |  |
| Human BOP1_R | CTAGGTGAAGAGGCGGACAGTCCCG |  |  |
| Human pmiRNA1-XbaI-HA-BOP1-F | CATAGAAGATTCTAGAGCC ACC ATG TACCCATACGACGTCCCAGACTACGCT GCGGGTTCGCGGGGTGCGG |  |  |
| Human pmiRNA1-Not1-BOP1-R | AGATCCTTCGCGGCCGC CTAGGTGAAGAGGCGGACAGTCCCG |  |  |
| P3 ITS1-5'Bio | /5Biosg/CCTCGCCCTCCGGGCTCCGGGCTCCGTTAATGATC | Northern Blot |  |
| P4 ITS2-5'Bio | /5Biosg/CTGCGAGGGAACCCCCAGCCGCGCA | Northern Blot |  |
